# Phosphorylation Changes SARS‐CoV‐2 Nucleocapsid Protein's Structural Dynamics and Its Interaction With RNA

**DOI:** 10.1002/prot.26842

**Published:** 2025-05-15

**Authors:** Stefan Loonen, Lina van Steenis, Marianne Bauer, Nikolina Šoštarić

**Affiliations:** ^1^ Department of Bionanoscience Kavli Institute of Nanoscience Delft, Delft University of Technology Delft HZ the Netherlands

**Keywords:** intrinsically disordered proteins, molecular dynamics, nucleocapsid protein, phosphorylation, protein–nucleic acid interactions, SARS‐CoV‐2

## Abstract

The SARS‐CoV‐2 nucleocapsid protein, or N‐protein, is a structural protein that plays an important role in the SARS‐CoV‐2 life cycle. The N‐protein takes part in the regulation of viral RNA replication and drives highly specific packaging of full‐length genomic RNA prior to virion formation. One regulatory mechanism that is proposed to drive the switch between these two operating modes is the phosphorylation state of the N‐protein. Here, we assess the dynamic behavior of non‐phosphorylated and phosphorylated versions of the N‐protein homodimer through atomistic molecular dynamics simulations. We show that the introduction of phosphorylation yields a more dynamic protein structure and decreases the binding affinity between the N‐protein and RNA. Furthermore, we find that secondary structure is essential for the preferential binding of particular RNA elements from the 5′ UTR of the viral genome to the N‐terminal domain of the N‐protein. Altogether, we provide detailed molecular insights into N‐protein dynamics, N‐protein:RNA interactions, and phosphorylation. Our results corroborate the hypothesis that phosphorylation of the N‐protein serves as a regulatory mechanism that determines N‐protein function.

## 1 Introduction

The severe acute respiratory syndrome coronavirus 2 (SARS‐CoV‐2) caused a global pandemic in 2019, and is still present to this day [1]. The SARS‐CoV‐2 viral life cycle comprises several stages: after binding to human host cells [2], the virus replicates and assembles into new virions inside the host cell [3, 4]. A SARS‐CoV‐2 virion consists of four structural proteins (spike, envelope, membrane, and nucleocapsid), a positive‐sense single‐stranded genomic RNA (gRNA) molecule, and a lipid envelope [5, 6]. Inside the virion, the nucleocapsid protein (N‐protein) and the gRNA form ribonucleoprotein complexes (RNPs), which organize into an ordered crystalline‐like “eggs‐in‐a‐nest” shaped assembly [5, 7]. Virions contain a remarkably low number of RNA molecules in the viral envelope, other than the gRNA [8], indicative of highly specific interactions between the N‐protein and the gRNA, as well as an ordered packaging mechanism [3, 9, 10, 11, 12]. Additionally, the N‐protein engages in a variety of non‐specific interactions with a multitude of interacting partners during the replicative phase of the viral infection cycle [4, 13, 14, 15, 16, 17, 18, 19, 20, 21, 22, 23, 24].

**FIGURE 1:**
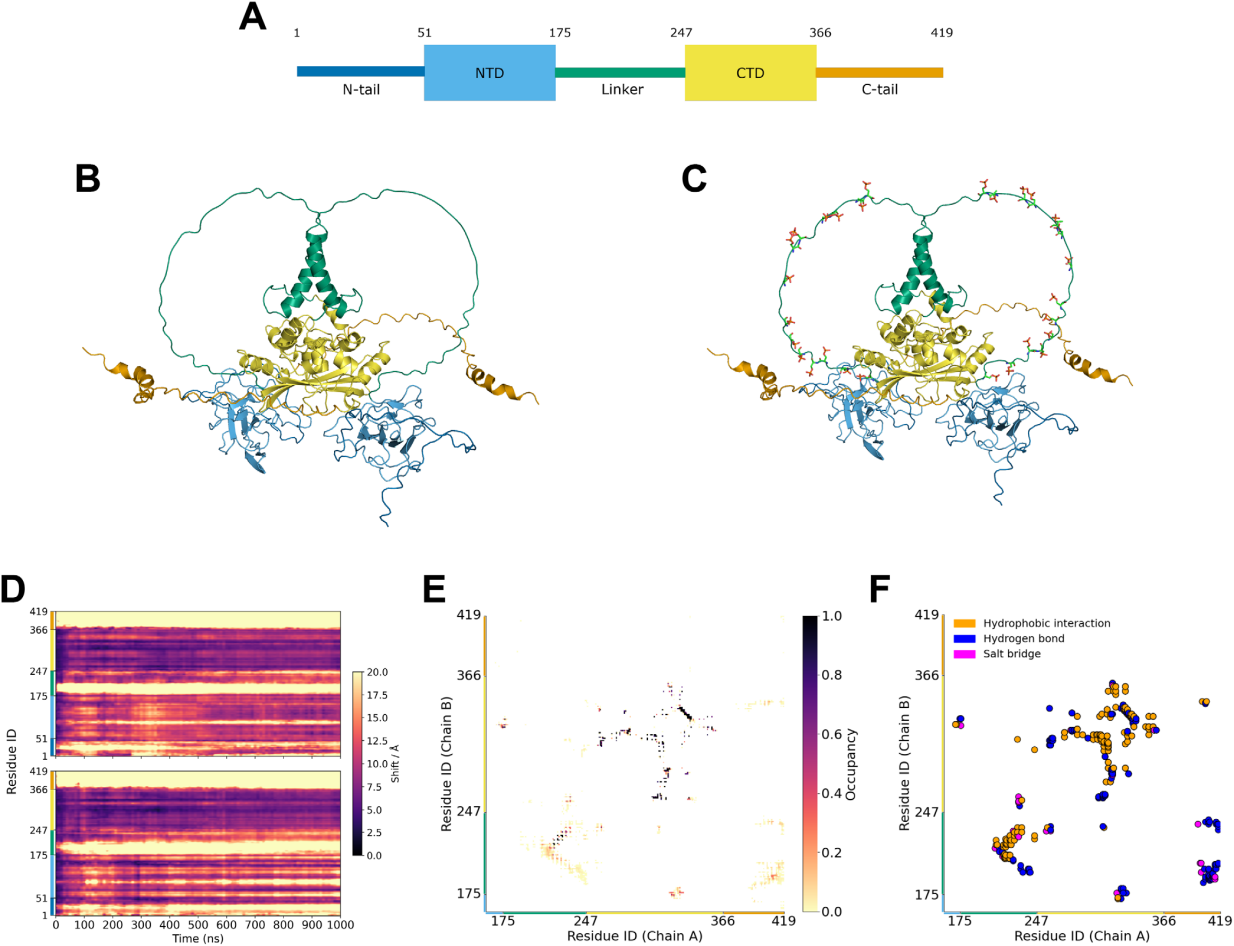
General insights into N‐protein structure and dynamics. (A) Scheme of the N‐protein regions: the N‐tail (dark blue, residues 1–50), N‐terminal domain (blue, 51–174), linker region (green, 175–246), C‐terminal domain (yellow, 247–365), and the C‐tail (orange, 366–419). (B) Cartoon representation of the N‐protein (Uniprot ID: P0DTC9) homodimer structure as predicted by AlphaFold Multimer. The regions are colored according to the legend shown in (A). (C) Phosphorylated version of the N‐protein structure, with the phosphorylated residues (licorice representation) corresponding to serine in positions 176, 180, 183, 184, 186, 188, 190, 194, 197, 201, 202 and 206, and threonine in positions 198 and 205. The added phosphogroups each carry a −2 charge. (D) Trajectory map generated using the TrajMap Python suite [26]. The shift of the backbone atoms of non‐phosphorylated N‐protein is calculated, for each residue and at each time point in a trajectory, with respect to the atom's initial position. The shading of a cell indicates the magnitude of the shift, with a dark color (black) corresponding to a low value and a light color (yellow) to a high value. We take the average over three replicates and produce two heatmaps, corresponding to both protomers that constitute a dimer. In the resulting figure stretches of yellow shading indicate a high deviation from the initial reference structure. (E) Intermolecular contact map generated using the Conan Python suite [27]. Contacts are shown between chain A and chain B of the non‐phosphorylated N‐protein homodimer. For an interaction we take a cutoff distance of 0.4 nm and a cutoff occupancy of 10%. We plot the average over three replicates and do not show the first 160 residues to highlight measured contacts. The shading corresponds to the average occupancy of a particular contact. We recognize residues in the CTD which are known to mediate dimerization, and thus are expected to be in contact as well as contacts between the alpha helices of the linker domain. (F) Interaction types of the contact plotted in (E). To generate the type of interaction the protein pdb was parsed into Conan which, based on the residue types, assigns an interaction type. The dimerization is mainly mediated through hydrophobic interactions and hydrogen bonds.

An N‐protein monomer consists of 419 amino acids, which can be grouped into five distinct regions [25] (Figure 1A). Two of these regions fold into domains and have their structure resolved through x‐ray crystallography [28, 29]. The other regions consist of two flexible tails (N‐ and C‐terminal) and a flexible linker that connects the two folded domains. In solution, the N‐protein exists predominantly as a dimer, where dimerization of two monomers is mediated by the formation of a stable beta sheet between two C‐terminal domains [30, 31]. There is a host of in vitro and in vivo reports on how the N‐protein functions and how its interaction with RNA changes as a result of structural domain knockouts in varying experimental conditions [12, 17, 24, 25, 30, 32, 33]. However, the full‐length 3D structure of the N‐protein homodimer has not been experimentally resolved.

The N‐protein can be post‐translationally modified (PTM) at several serine and threonine phosphosites, as detected by mass spectrometry [34, 35]. These phosphosites are most abundant in the serine‐rich (SR) region of the linker. Early in the infection, the N‐protein pool in the cytosol is heavily phosphorylated by host kinases, which are likely able to switch the N‐protein linker domain from being non‐phosphorylated to fully phosphorylated in a short time‐frame by employing so‐called phosphorylation cascades [34, 35, 36]. Phosphorylation of the N‐protein's SR‐region is proposed to be a key regulatory mechanism that determines N‐protein function throughout the infection cycle [17, 32, 36, 37, 38, 39, 40, 41, 42]. The N‐proteins that form the RNPs in an assembled virion are non‐phosphorylated [36], indicating that the N‐protein plays different functional roles in different PTM states. Some research suggests that phosphorylation of the N‐protein diminishes the interaction between N‐protein and RNA [32, 41, 42]. In contrast, studies on the N‐protein in other coronaviruses found that phosphorylation does not change binding to RNA, but instead affects the N‐proteins' ability to form larger, potentially multimerized clusters [43, 44]. Indeed, phosphorylation of the N‐protein has been suggested to lead to lower viscosity condensates with RNA in in vitro studies of the protein compared to condensates without phosphorylation, implying a difference in either N‐protein:RNA or N‐protein:N‐protein interactions. Thus, establishing how phosphorylation structurally affects the N‐protein conformation and its interaction with different RNA types relevant for both in vitro and in vivo studies is an important question which we address in this work.

Reports on N‐protein:RNA liquid–liquid phase separation often rely on polyU and polyA in experiments [25, 45, 46]. Furthermore, the N‐protein was shown to have a preferred binding affinity for specific secondary structures found in the 5′ UTR of the viral genome [47]. This region contains a conserved secondary structure with a high degree of hairpins, as well as the leader transcription regulatory sequence (TRS‐L) motif, which is crucial for template switching [11, 48, 49].

We employ atomistic molecular dynamics simulations to better understand the impact of phosphorylation on the dynamic behavior of phosphorylated and non‐phosphorylated versions of the N‐protein homodimer. Although there are in silico reports modeling the N‐protein, most of these studies either simulate truncated versions of the N‐protein monomer, or use coarse‐grained approaches [11, 21, 25, 28, 31, 50, 51, 52, 53]. We show that the introduction of phosphorylation yields a protein structure with more conformational flexibility. Furthermore, to investigate how the N‐protein interacts with RNA, we introduce four types of RNA into the simulation: polyU, polyA, and two specific 5′ UTR elements, consisting of stem‐loop 2 and 3 (SL2SL3), and stem‐loop 4 with an extended region (SL4ext). We find that non‐phosphorylated N‐protein forms a compact and equilibrated complex with all respective RNA molecules. Phosphorylation of the N‐protein destabilizes these complexes. Our results support the hypothesis that phosphorylation within the N‐protein pool serves as a regulatory mechanism, fine‐tuning N‐protein function throughout the viral infection cycle. Moreover, we emphasize the importance of secondary structure of the RNA in N‐protein:RNA interactions by showing that the SL4ext molecule preferentially binds the NTD only in its folded state. We envision that our atomistic simulations of the full‐length N‐protein homodimer can be used as a reference point for future in silico analysis and to guide experimental work.

## 2 Results

### 2.1 General Insights Into N‐Protein Structure and Dynamics

The SARS‐CoV‐2 N‐protein can be roughly divided into five different regions (Figure 1A): the N‐tail (residues 1–50), the N‐terminal domain (NTD, 51–174), the serine‐rich linker (175–246), the C‐terminal domain (CTD, 247–365), and the C‐tail (366–419). The N‐tail, linker, and C‐tail are predicted to be (partially) disordered [25]. We obtained the starting homodimer structure for the molecular dynamics simulations by submitting the amino acid sequence of the N‐protein (UniProt: P0DTC9) to AlphaFold Multimer [54, 55] (AF). We validated the five top‐ranked structures by aligning their CTD and NTD with known crystal structures of these domains (PDB: 6WZO, 6VYO) (Figure S1). The predicted structures obtained good alignments, with all RMSD values being below 1 Å (Table S1). The structures thus mainly differ in their disordered regions, which we expect to have a high degree of conformational flexibility. Therefore, from the five top‐ranked structures, we selected the one that reached the lowest energy value after steepest descent energy minimization (Figure 1B; Table S1). To prepare the phosphorylated N‐protein structure, we chose to phosphorylate all experimentally identified phosphosites in both linker regions of the N‐protein homodimer to compare two previously identified PTM states of the N‐protein (non‐phosphorylated and fully phosphorylated) [35] (Figure 1C). We performed three 1 μs atomistic molecular dynamics simulations for both the non‐phosphorylated and phosphorylated N‐protein homodimer, amounting to a total simulation time of 3 μs per system (Table 1).

**TABLE 1:** Overview of the produced simulations.

N‐protein state	RNA sequence	Color code	Production time
Non‐phosphorylated	No RNA	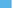	3 × 1 μs
Phosphorylated	No RNA	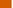	3 × 1 μs
Non‐phosphorylated	PolyA	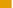	1 μs
Non‐phosphorylated	PolyU	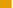	1 μs
Non‐phosphorylated	SL2SL3	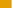	1 μs
Non‐phosphorylated	SL4ext	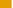	1 μs
Phosphorylated	PolyA	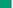	1 μs
Phosphorylated	PolyU	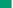	1 μs
Phosphorylated	SL2SL3	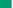	1 μs
Phosphorylated	SL4ext	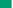	1 μs
NTD	no RNA	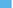	3 × 1 μs
NTD	PolyA	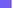	3 × 1 μs
NTD	PolyU	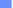	3 × 1 μs
NTD	SL2SL3 stretched	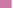	3 × 1 μs
NTD	SL2SL3 folded	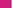	3 × 1 μs
NTD	SL4ext stretched	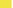	3 × 1 μs
NTD	SL4ext folded	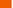	3 × 1 μs

To obtain first insights into the N‐protein homodimer, we evaluate the movement of the backbone atoms of the non‐phosphorylated N‐protein over a trajectory, with respect to each atom's initial position (Figures 1D and S2A). We observe a rapid deviation from the initial position of the C‐tail, parts of the linker region, and parts of the N‐tail, the regions that are predicted to be disordered and should therefore be more flexible. A specific stretch of residues in the NTD is also substantially fluctuating in nearly all trajectories and corresponds to a beta coil (residues 88–111) pointing outwards from the NTD. The beta coil is known to interact with RNA, possibly serving as a mediator for initial RNA binding [28, 52]. The remainder of the NTD, as well as the entire CTD domain, exhibit reduced dynamics throughout the majority of the trajectories when compared to the disordered regions.

We investigated the intermolecular interactions in the N‐protein homodimer through a geometric‐based contact map (Figure 1E). Dimerization of the two protomers is mediated by a stable beta sheet formed by two CTD beta coils of both protomers (residues 330–340). The stability of the beta sheet is highlighted by the 100% occupancy observed throughout all replicates. Other regions contributing to the dimerization interaction include alpha‐helices in the CTD and the alpha‐helix in the linker domain. The interaction between the two alpha‐helices in the linker domains is consistent with recent experimental findings that point to this same interaction [56]. The majority of interactions consist of hydrophobic interactions and hydrogen bonds (Figure 1F).

### 2.2 Phosphorylation of the N‐Protein Increases Its Conformational Flexibility

**FIGURE 2:**
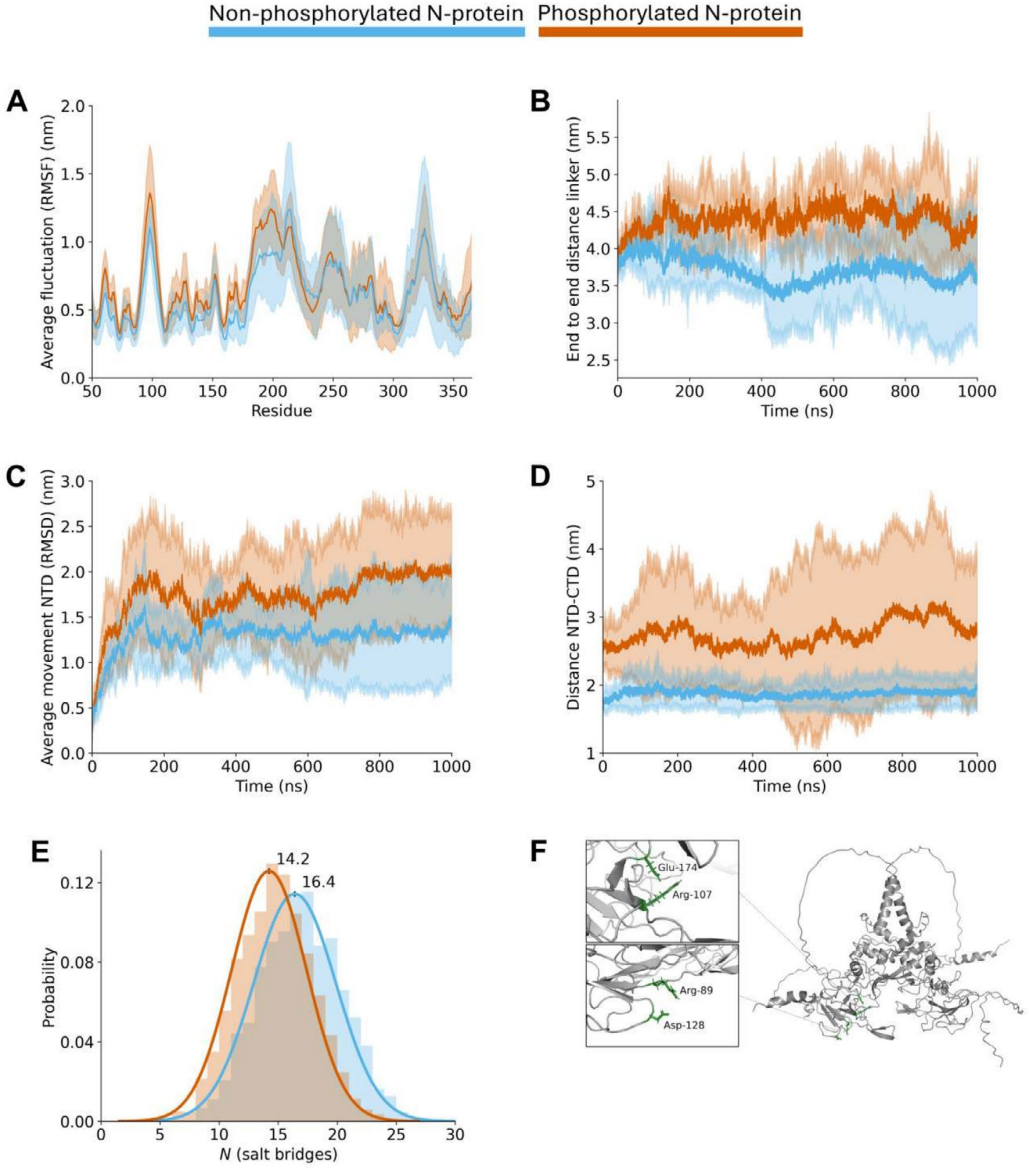
Phosphorylation of the N‐protein increases its conformational flexibility. In this figure, data for the non‐phosphorylated N‐protein is shown in blue, and data for the phosphorylated protein in orange. The plotted data include an average of two protomers for each dimer and of three separate 1 μs simulations, where the calculated mean is shown as a solid line encompassed by a standard deviation sized shading. (A) The root mean square fluctuation of the protein backbone atoms, averaged per residue. There are increased fluctuations in the NTD domain (51–174) and linker region (175–246) of the phosphorylated N‐protein. The panel does not show the flexible tails (residues 1–50 and 366–419). The panel including the tails can be found in the Supporting Information (Figure S4C). (B) End‐to‐end distance of the linker region as measured by *gmx polystat*. The distance is larger in the phosphorylated version of the N‐protein. (C) The average movement of the backbone atoms of the NTD domain of the N‐protein as measured through the root mean square deviation. The starting structure of the whole protein is taken as a reference for the calculation, in order to capture the movement of the NTD relative to the whole protein. The NTD domain moves more when the N‐protein is phosphorylated. (D) The distance is shown between the center of mass of the NTD and the residue in the CTD that is closest to the NTD at the initial time point of the trajectory. A representation of this distance can be found in the Supporting Information (Figure S5). The distance increases, on average, to a larger extent when the N‐protein is phosphorylated. (E) Probability density graph for the number of salt‐bridges in the simulations. The histograms show the raw counts (*N* = 3000) of the number of salt‐bridges across time points. A Gaussian fit is shown based on the mean and standard deviation. The phosphorylated N‐protein forms 1.8 salt‐bridges less, on average. (F) Cartoon representation of the N‐protein homodimer, colored in gray. The insets show two specific salt‐bridges in licorice representation, Arg89‐Asp128 and Arg107‐Glu174 (green), that exhibit a high occupancy in the non‐phosphorylated version of the protein and a low occupancy in the phosphorylated version.

As we are interested in the effect of phosphorylation on N‐protein dynamics, we compare the flexibility between non‐phosphorylated and phosphorylated N‐protein. To this end, we calculated the root mean square fluctuations (RMSF) over a trajectory for each residue's backbone atoms, averaging over both protomers and the three replicates (Figures 2A and S3C). When the N‐protein is phosphorylated, the NTD (51–174) and linker region (175–246) fluctuate more compared to the N‐protein in its non‐phosphorylated state. The remaining regions in the protein exhibit smaller differences in the RMSF, as well as in the backbone atom shift (Figure S2B). The radii of gyration of the N‐protein in both its non‐phosphorylated and phosphorylated state evolve in a similar manner (Figure S3E,F), as do the end‐to‐end distances of the protomers (Figure S3G).

We characterized the movement of the NTD and linker through four spatial measures. We observe that the end‐to‐end distance of the linker region (Gly175‐Val246) increases upon phosphorylation and becomes on average 0.8 nm longer in the phosphorylated state (Figure 2B). In addition, we find that the root mean square displacement (RMSD) of the NTD backbone atoms with respect to their starting positions is, on average, 0.65 nm higher when the N‐protein is phosphorylated (corresponding to 50% of the average RMSD of non‐phosphorylated NTD backbone atoms) (Figure 2C). In order to investigate what might cause the increased length of the linker, we calculated the distance between the closest residues of the NTD and the CTD at each time point. This measures an effective minimal distance between the NTD and CTD surfaces, and we observe that this minimal distance is maintained at approximately 0.45 nm throughout the simulations. This implies that when an NTD moves, it generally moves tangentially along the CTD surface (Figures S3H and S4A). Thus, we finally examined this movement of the NTD by computing the distance between the center of mass of the NTD and the initial closest residue of the CTD (Figures 2D and S4B). In the simulations of the phosphorylated N‐protein, we find a large increase in this distance, reaching a maximum average value of 3 nm, as well as a significant variation in the distance, which ranges between 1 and 4.5 nm. In contrast, the center of mass of the NTD in the non‐phosphorylated N‐protein remains significantly closer to the initial closest residue of the CTD, with a maximum average distance of 2 nm and a range between 1.5 and 2.5 nm. When we measure the angle spanned by the centers of mass of the NTD‐CTD‐NTD, we observe that the angle is more variable for the phosphorylated N‐protein (Figure S3I). Taken together, these measures show increased dynamics of the phosphorylated N‐protein homodimer, which are most pronounced in the NTD and linker regions.

The increased dynamics of the NTD and linker region could, at least partially, be explained by the loss of salt bridges. We see that on average less salt bridges form within the phosphorylated protein, with respect to the non‐phosphorylated version (14.2 compared to 16.4 salt bridges) (Figure 2E). After analyzing which salt bridges were present in more than 50% of each trajectory (Table S2), we found that there is exactly one salt bridge, Arg107‐Glu174, that is present in all three non‐phosphorylated replicates but not in any of the replicates of phosphorylated N‐protein. This salt bridge seems to be a stabilizing connection between the NTD and the central CTD structure (Figure 2F). We hypothesize that the increase in NTD movement found in the phosphorylated simulations can largely be attributed to the loss of this particular salt bridge. The increased fluctuations within the NTD can be attributed to the loss of another salt bridge, Arg89‐Asp128 (Figure 2F). In two of the three non‐phosphorylated replicates, this salt bridge is present, but not in any of the phosphorylated simulations.

Finally, we assess the overall stability of the homodimer by comparing, between the non‐phosphorylated and phosphorylated N‐protein, the free energies of binding between the protomers [57] (see Methods). Phosphorylation has a destabilizing effect on the dimerization interaction, as indicated by a positive ΔΔ *G* ($\Delta \Delta G=\Delta G_{\mathrm{Ph}}-\Delta G_{\text{nonPh}}=32.2\;\text{kcal}/\mathrm{mol}$; Table S3). When comparing the geometric based contact maps between non‐phosphorylated (Figure 1E) and phosphorylated (Figure S5A) N‐protein, we observe that the main interactions that are lost lie in the linker domain and consist of hydrogen bonds (Figures 1F and S5B). To further quantify which residues contribute differentially to the binding energy between the protomers, we examine residue specific contributions to the free energy of binding. Residues in the CTD (residues 247–365) are most essential for the stability of the homodimer in both PTM states (Figure S5C,D). When we compare the non‐phosphorylated and phosphorylated per‐residue contributions (ΔΔ *G*), we find that the contribution to the binding affinity for multiple residues outside of the linker region is affected by phosphorylation (Figure S5E). This indicates that the influence of phosphorylation is not limited to short‐range effects, but can also induce long‐range conformational changes, that affect binding affinity.

Altogether, we conclude that phosphorylation of the serines and threonines in the linker region of an N‐protein homodimer leads to a higher flexibility of the protein, especially in the NTD and linker region. We suggest that this increased flexibility is, at least partially, explained by reduced hydrogen bonding between the linker domains and the loss of a particular salt bridge (Arg107‐Glu174), which can induce long‐range conformational changes.

### 2.3 N‐Protein:RNA Binding Is Diminished by Phosphorylation

**FIGURE 3:**
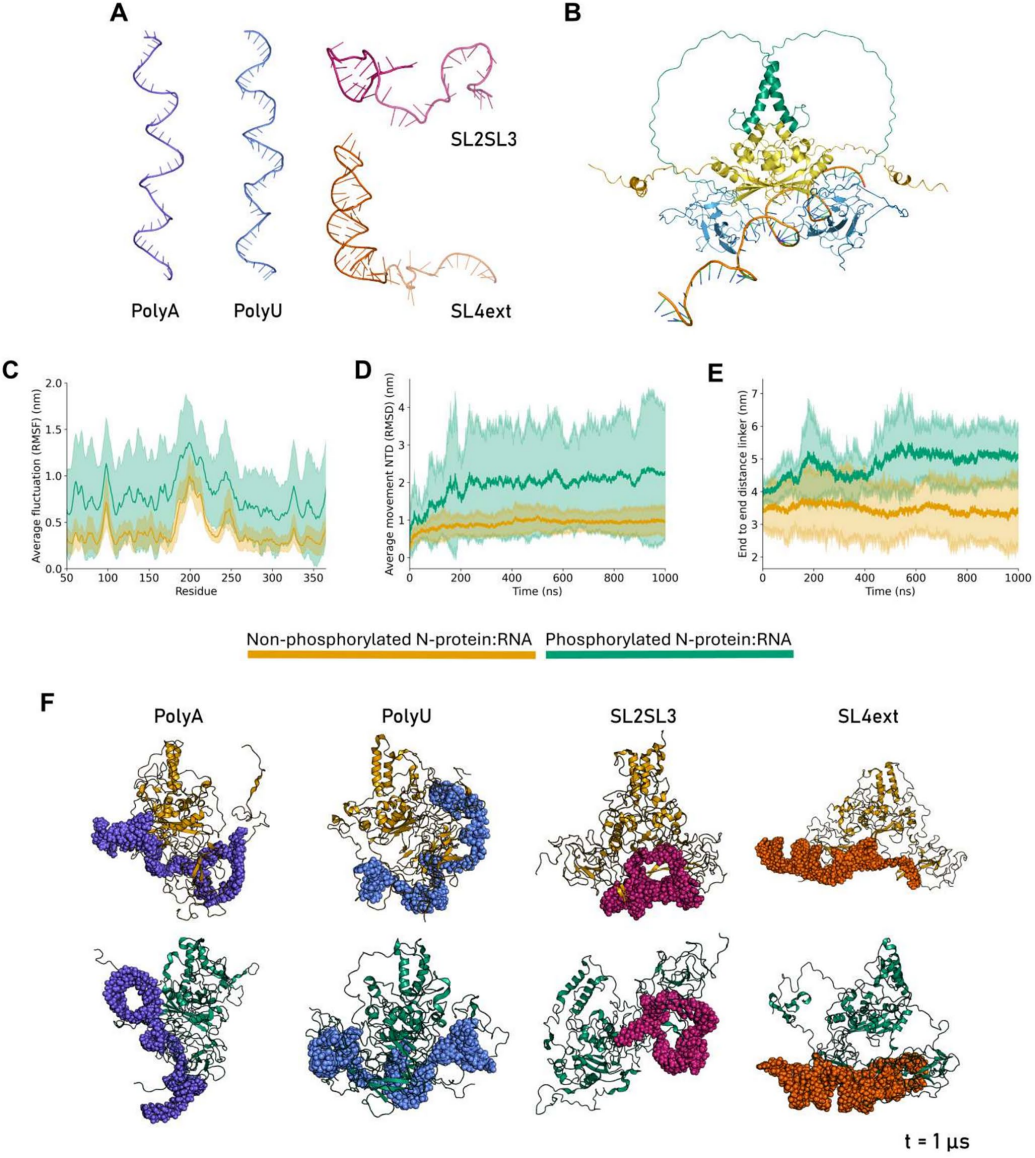
N‐protein:RNA binding is diminished by phosphorylation. (A) The RNA molecules used in the simulations, shown in cartoon representation. For SL2SL3 and SL4ext the SL3 and ext. regions have a lower opacity. (B) The docking output of HADDOCK [58], showing a polyU RNA docked to residue Arg107 of the N‐protein. (C) The average RMSF of the protein backbone atoms is shown for non‐phosphorylated (yellow) and phosphorylated (green) N‐protein. The error shading shows the standard deviation between four simulations with varying RNA molecules. The panels do not show the flexible tails (residues 1–50 and 366–419). The panels including the tails can be found in the Supporting Information (Figure S6A). (D) The average movement of the backbone atoms of the NTD domain of the N‐protein as measured through the root mean square deviation, measured for all four RNA types. The starting structure of the whole protein is taken as a reference for the calculation, in order to capture the movement of the NTD relative to the whole protein. The NTD domain moves more when the N‐protein is phosphorylated. (E) End‐to‐end distance of the linker region as measured by *gmx polystat*. The distance is, on average, increased when the N‐protein is phosphorylated. (F) The endpoints of each simulation are shown. The RNA molecules are colored according to the color code introduced in (A) and shown using the spheres representation of PyMOL. The protein is shown in cartoon representation and colored according to phosphorylation state.

Seeing as the N‐protein can have different binding interactions with RNA, we set out to investigate how phosphorylation attenuates the interaction between N‐protein and RNA. To this end, we tested four types of RNA: polyU and polyA (both 50 nt), which are frequently used in in vitro experiments, as well as two regions of the 5′ UTR, namely a combination of stem‐loop 2 and 3 (SL2SL3, 41 nt), which contains the leader TRS motif, as well as stem‐loop 4 with an extended region (SL4ext, 67 nt), which was shown to preferentially bind the N‐protein NTD [47] (Figure 3A; Table S4). We chose the polyU and polyA molecules for their frequent use in experimental setups [59] and SL2SL3 and SL4ext for their biological relevance. We docked the RNA molecules to experimentally identified RNA binding sites on the N‐protein [28] (Arg92 and Arg107, Figure 3B). With these starting structures, we produced 1 μs atomistic molecular dynamics trajectories of both the non‐phosphorylated and phosphorylated N‐protein, in combination with the aforementioned RNA types, yielding a total of 8 μs of simulation time (Table 1).

First, we calculated the free energy of binding between the N‐protein and RNA. Non‐phosphorylated N‐protein forms a stable complex with all tested RNA molecules over the duration of the simulation. Phosphorylation destabilizes this interaction, as indicated by a positive ΔΔ *G* across all RNA systems (Table 2). The destabilization is further highlighted by significantly increased fluctuations of the protein backbone (Figure 3C), a greater average movement of the NTD domains (Figure 3D), and extension of the linker domains (Figure 3E) in the phosphorylated systems. The radius of gyration of the protein is smaller for non‐phosphorylated N‐protein (Figure S6B), reflecting a more compact N‐protein:RNA complex, and the RMSD of the full N‐protein dimer is higher when phosphorylated (Figure S6C). Still, all RNA molecules equilibrate in both the non‐phosphorylated and phosphorylated N‐protein simulations indicated by a stabilization of the RMSD (Figure S6D), and a general decrease in radius of gyration (Figure S6E) across the tested RNA species.

**TABLE 2:** MMGBSA results for the N‐protein:RNA simulations.

RNA sequence	Non‐phosphorylated Δ*G* (kcal/mol)	Phosphorylated Δ*G* (kcal/mol)	Ph–nonPh ΔΔ*G* (kcal/mol)
PolyA	−223.98	−91.04	132.94
PolyU	−589.11	−289.24	299.87
SL2SL3	−360.49	−98.26	262.23
SL4ext	−385.49	−70.96	314.53

We further investigated whether N‐protein:RNA interactions might be sequence‐ or secondary structure‐dependent by evaluating the end‐points of each simulation (Figure 3F). We qualitatively observe that the N‐protein structure remains similar between the RNA simulations when the N‐protein is not phosphorylated. In the simulations containing phosphorylated N‐protein, we observe a larger variation in the protein structure between the simulations with different RNA systems. Phosphorylation attenuates the N‐protein:RNA interaction, where the degree of attenuation depends on the type of RNA. To further quantify differences between the RNA simulations, we compared the hydrogen bonding, mapped intermolecular interactions, and decomposed the free energy of binding between the N‐protein and RNA into per‐residue contributions.

Phosphorylation diminishes the number of hydrogen bonds formed between the N‐protein and RNA when in complex with the polyA (Figure S7A), the SL2SL3 (Figure S9A), and the SL4ext (Figure S10A) molecules, while the number is comparable for the polyU (Figure S8A). The majority of the residues that contribute differentially to binding, when comparing PTM states, lie in the beta coil of the NTD region (residue 88–111), which directly interacts with the RNA (Figures S7B, S8B, S9B, and S10B). There are residues that have an increased binding affinity as a result of phosphorylation and stabilize (−), or have a decreased binding affinity and destabilize (+) the N‐protein:RNA interaction. When taking a cutoff of ∣ΔΔG∣>2.5 kcal/mol we find that these are mostly positively charged residues such as arginines or lysines (**polyA**: +Arg92, +Arg93, −Arg95, +Lys100, +Lys102, +Leu104, +Lys127. **polyU**: +Arg89, −Arg95, −Arg100, +Asp103, −Arg107, −Arg149. **SL2SL3**: +Arg93, +Lys100, +Lys127, +Asp128, +Asp144. **SL4ext**: −Arg88, +Arg92, −Arg93, +Arg95, −Arg100, −Lys102, +Glu118 (chain A), −Glu118 (chain B), −Lys127, +Asp128). The binding affinity for several residues that are part of the CTD is also affected by phosphorylation (**polyA**: −Arg276, **polyU**: +Glu280, −Arg319, +Glu323, −Thr332, +Asp358, **SL2SL3**: +Arg319, +Lys369, −Asp371, **SL4ext**: +Asp358, −Lys361). The impact of phosphorylation is directly showcased by the SL2SL3 simulation, where the negatively charged phosphorylated Ser176 in the linker region is actively repelling the negatively charged RNA backbone.

Lastly, intermolecular contact maps between the N‐protein and RNA give insight into the contacts each RNA molecule makes with the N‐protein. We observe that the polyA (Figure S7C,D), polyU (Figure S8C,D), and SL2SL3 (Figure S9C,D) RNA molecules form contacts with both non‐phosphorylated and phosphorylated N‐protein with most of their nucleotides. In contrast, the contacts made by SL4ext (Figure S10C,D) are primarily confined to its extended region (nucleotides 48–64), consistent with recent experimental studies highlighting the importance of this region for the high binding affinity between the NTD and SL4ext [47].

In conclusion, the N‐protein is able to form stable complexes with a variety of RNA molecules. These complexes are significantly destabilized by phosphorylation, but the effect of phosphorylation on the dynamical behavior and stability of the N‐protein:RNA complex depends on the type of RNA involved. We make these observations using single 1 μs replicates for each RNA type tested in either non‐phosphorylated or phosphorylated N‐protein conditions. To further disentangle the effects of sequence and secondary structure on N‐protein:RNA interactions, we performed additional simulations using only the NTD with the various RNA molecules.

### 2.4 NTD:RNA Interactions Depend on RNA Sequence and Structure

**FIGURE 4:**
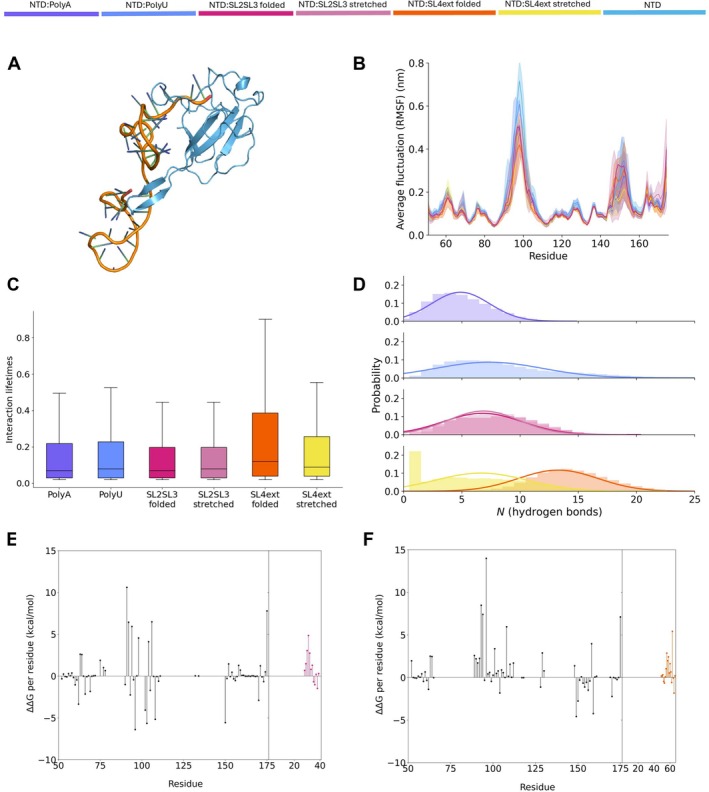
NTD:RNA interactions depend on RNA sequence and structure. (A) Cartoon representation of the NTD (residues 51–175) as isolated from the AlphaFold 2 dimer with PyMOL, docked to SL2SL3 folded RNA using HADDOCK. (B) The average RMSF of the NTD's backbone atoms, for each different RNA type, colored as in the legend above, over the triplicates with standard deviation is shown with error shading. (C) The lifetimes of each interaction in a simulation between the NTD and RNA are plotted for each simulation. For an interaction we take a cutoff distance of 4 Å and a cutoff lifetime of 0.01. The boxes in the boxplot are bounded by the first and third quartile, indicating that SL4ext in its folded state forms interactions that are on average longer lived when compared to the other RNA molecules. (D) Probability density graph for the number of hydrogen bonds in the triplicates. The histograms show the counts of hydrogen bonds per timepoint. A Gaussian fit is shown based on the mean and standard deviation. We note here that for stretched SL4ext, we find many hydrogen bonds at the zero bin due to the disassociation of the RNA from the NTD in one of the replicates. This replicate is not used for generating the Gaussian fit. The change in binding energy between the NTD and (E) SL2SL3 or (F) SL4ext comparing folded and stretched structure. There are two subplots; the first shows the residues of the NTD, and the second shows the residues of the respective stem‐loop. The ΔΔG is calculated by subtracting the stretched ΔG from the folded ΔG ($\Delta \Delta G=\Delta G_{\text{folded}}-\Delta G_{\text{stretched}}$).

The N‐protein homodimer interacts with the RNA through its NTD domains [28]. We thus further evaluated different RNA structures and their binding to the isolated NTD, which as a smaller system allows for more simulations in a shorter time period. The polyA, polyU, as well as the folded SL2SL3 and SL4ext sequences were docked to a single NTD domain (Figure 4A). Additionally, we performed simulations in which we docked SL2SL3 and SL4ext to the NTD in a stretched‐out conformation, such that we could directly investigate the effect of secondary structure on the NTD:RNA interaction. Lastly, we also generated control trajectories of the NTD without RNA. Simulations with six different RNA molecules docked to Arg92, and the NTD without RNA, were produced in triplicates, each with a 1 μs duration, yielding a total of 21 μs of simulation time (Table 1).

We observe that all RNA types can stably bind the NTD, which can be seen in the endpoints of the simulations (Figure S11). The polyA and polyU show variable binding modes across the triplicates, in which the orientation of the RNA molecule with respect to the NTD varies. We further note that the stretched versions of the stem‐loop simulations start folding into secondary structures over the course of the trajectory, and their initially folded counterparts stably bind the NTD. Notably, one replicate of the stretched SL4ext simulation completely dissociates from the NTD during the trajectory.

To quantify the stability of the different NTD:RNA interactions we compared the binding affinities between the NTD and respective RNA molecules. Stable RNA:NTD interaction is confirmed by the negative average binding energy (Table 3). Folding of the RNA stabilizes the interaction of the stem‐loops with the protein domain, indicated by the negative ΔΔ *G* when subtracting the stretched measurements from the folded measurements. This is especially true for SL4ext, demonstrating the importance of its secondary structure when interacting with the NTD.

**TABLE 3:** MMGBSA results for the NTD:RNA simulations.

RNA sequence	Stretched RNA Δ*G* (kcal/mol)	Folded RNA Δ*G* (kcal/mol)	Folded—Stretched ΔΔ*G* (kcal/mol)
PolyA	−48.35	—	—
PolyU	−88.29	—	—
SL2SL3	−81.17	−82.22	−1.05
SL4ext	−52.02	−128.90	−76.88

*Note:* The reported ΔG values are averaged over three replicates.

The decomposition of the binding free energy per residue shows stabilizing contributions from residues in the beta coil region of the NTD, and a few in the looping region between residues 145–158, both for SL2SL3 (Figure 4E) and SL4ext (Figure 4F). With the cutoff of ∣ΔΔ *G* ∣>2.5 kcal/mol, we identify residues that have a higher contribution to the NTD:RNA interaction energy upon RNA folding and stabilize (−) as well as residues that have a lower contribution upon folding and destabilize (+) RNA:NTD interaction (**SL2SL3**: +Glu62, +Arg88, +Arg92, +Arg93, +Arg95, +Lys100, +Arg107, +Asp128, −Thr148, −Arg149, +Ile157, −Val158, +Glu174. **SL4ext**: +Arg93, −Arg95, −Lys61, +Glu62, +Asp63, +Ala90, +Thr91, +Gly97, −Met101, −Lys102, +Asp103, +Ser105, +Arg107, +Arg149, +Lys169, +Glu174).

From the NTD's RMSF, we identify two regions showing differential behavior across the simulations. The first region stabilizes upon RNA binding and corresponds to the beta coil to which the RNA molecules are docked (residue 88–111). The folded SL4ext RNA structure reduces the fluctuations of this region the most (Figure 4B). The second region consists of residues 145–158 and represents a small loop that is stabilized in the context of the full N‐protein homodimer (Figure 2A). The RMSD of the NTD reflects the stability of the domain, is relatively low, and equilibrates quickly for all simulations (Figure S12A). The RMSD of the RNA C5 backbone atoms evens out as well, albeit at different absolute values (Figure S12B). The radii of gyration of the respective RNA molecules range between 1.6 (SL2SL3) and 3.9 nm (SL4ext) (Figure S12C). When measuring the intermolecular contacts, we find that the different RNA types interact with comparable regions in the NTD (Figure S13A–F). However, the lifetime of these interactions is on average longer for the folded SL4ext structure (Figure 4C), which is partially explained due to the number of hydrogen bonds formed between the NTD and RNA (Figure 4D). The folded SL4ext forms a high number of hydrogen bonds per time point, whereas polyA and SL2SL3 have a higher probability of finding a low number of hydrogen bonds. For polyU and stretched SL4ext, there is a broader distribution in the number of hydrogen bonds formed at a given time point.

Together, our results show that NTD:RNA interactions are driven both by sequence and structure. By comparing the different NTD:RNA systems, we see that the NTD preferentially binds to the folded SL4ext structure, in agreement with previous work on NTD:RNA binding affinities [47]. In this section, we identified residues contributing to this favored binding, highlighted the importance of the secondary structure of SL4ext for the binding affinity, and gave detailed insights into NTD:RNA interactions.

**FIGURE 5:**
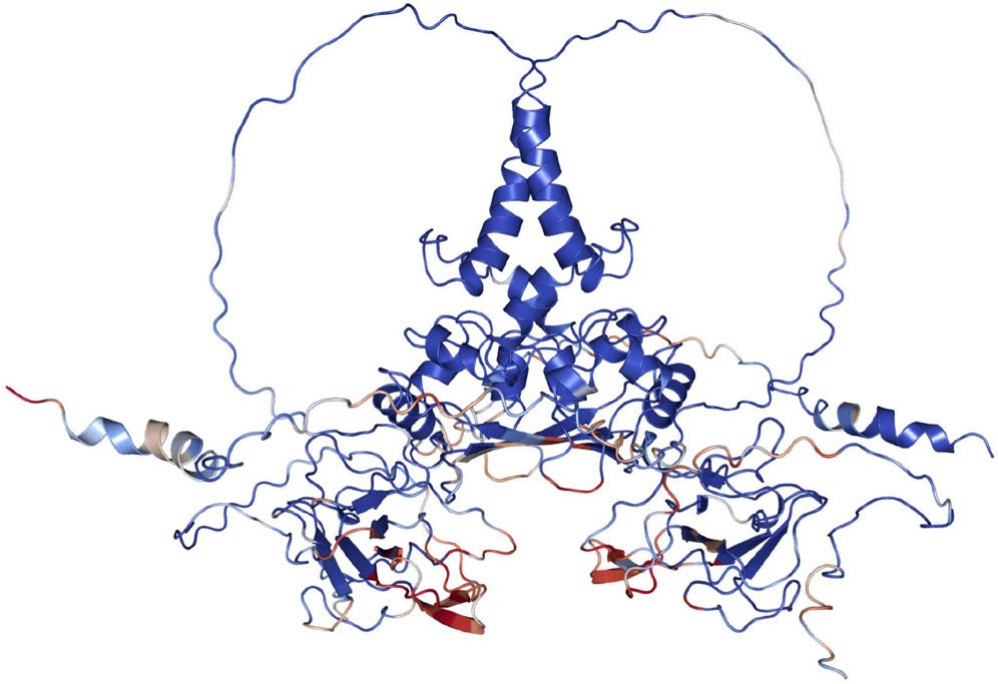
The N‐protein homodimer colored according to occupancy of N‐protein:RNA contacts. Contacts were measured using the Conan Python suite [27]. The color code corresponds to the maximum occupancy of a contact found between the different simulations with RNA. The darker red a residue is colored, the higher its maximum occupancy is. The beta coils in the NTD have a high occupancy, as expected.

Inspired by the contact maps, we return to the question of the full protein and the potential RNA binding pockets it might harbor. Altogether, the tested RNA molecules interact with a wide array of residues on the N‐protein. We summarize the contacts the RNA molecules make with the N‐protein in our simulations in Figure 5, where we colored the protein according to the lifetimes of occupancies. We observe that all five domains are able to form contacts with RNA, implying that there is not one single RNA binding pocket. Instead, each domain can bind RNA, but the binding affinity can be tuned by the RNA sequence, structure, length, as well as the PTM state of the N‐protein itself. This is in line with previous reports on N‐protein:RNA binding which identified multiple potential RNA binding pockets [24, 25, 30].

## 3 Discussion

The SARS‐CoV‐2 N‐protein contains several disordered regions and forms a homodimer in solution at physiologically relevant conditions [14, 30]. The spatial organization of the homodimer is challenging to resolve experimentally and can be affected by the protocol used to purify the N‐protein [60]. As such, a unifying view on N‐protein structure and function remains elusive. The N‐protein is able to discriminate between non‐specific interactions during the replication phase and highly specific packaging of the viral genomic RNA. A proposed regulatory mechanism governing N‐protein function is the degree of phosphorylation in the N‐protein pool [17, 38, 42], which is shown to vary over the course of an infection [35].

In this study we investigated the full‐length N‐protein homodimer through atomistic molecular dynamics simulations and evaluated the effect of phosphorylation on the structural dynamics of the N‐protein. We also explored N‐protein:RNA interactions and the effect of phosphorylation on these interactions. Lastly, we screened four different types of RNA in complex with the NTD domain of the N‐protein and explored the impact of sequence and secondary structure on the resulting interaction. Our results highlight an increased flexibility of the N‐protein structure in its phosphorylated state. We show that non‐phosphorylated N‐protein forms stable complexes with a variety of RNA molecules, which are destabilized by N‐protein phosphorylation. Finally, we disentangle the importance of RNA sequence and structure for binding to the N‐protein NTD and emphasize the importance of secondary structure of the SL4ext region in the 5′ UTR for its preferential binding to the N‐protein NTD.

The results presented here can be understood both on the scale of a larger ensemble of molecules, as well as on the scale of single molecules and their interactions. In vitro experiments have implicated the mechanism of liquid–liquid phase separation (LLPS) in packaging, which occurs across a range of N‐protein concentrations and RNA types. Generally, phosphorylation lowers the viscosity of N‐protein:RNA condensates, indicating that the interactions in such a condensate are weak and more transient [39]. We show, at the single molecule level that phosphorylation of the N‐protein yields a more flexible protein, which suggests a decreased viscosity at the ensemble level. On the cellular level, the packaged viral genome is thought to be organized in well‐ordered “eggs‐in‐a‐nest”‐based ribonucleoprotein complexes (RNPs) [5, 7]. Our findings could imply that phosphorylated N‐protein is not able to form RNPs that are stable enough to organize into these crystalline multimerized structures. This hypothesis can be further investigated through simulations which contain multiple N‐proteins and longer stretches of RNA.

We can also interpret our findings in the context of polymerase template switching, which occurs during transcription [61]. It was shown in vitro that the 5′ UTR region drives the formation of condensates with the N‐protein [9, 10]. We find that the secondary structure of SL4ext is important for its preferential binding affinity to the N‐protein, but that the secondary structure does not impact the binding between the N‐protein and SL2SL3. SL2SL3 contains the leader transcription regulatory sequence (TRS‐L) to which any of the body transcription regulatory sequences (TRS‐B) are matched during template switching [61]. Potentially, SL4ext serves as a hook for the N‐protein, which then helps bring the RNA‐dependent RNA polymerase (RdRp) close enough to match the two complementary TRS motifs. This hypothesis is a slightly nuanced version of the initially proposed model for N‐protein regulated polymerase template switching in viral replication of coronaviruses, in which it was proposed that the N‐protein directly binds the TRS‐L motif [62].

The strong binding between SL4ext and the N‐protein can also point to the selectivity in genome packaging which is observed in vivo [8]. An RNA element structurally resembling SL4ext, termed P3, is hypothesized to be the packaging signal to discriminate between gRNA and sgRNAs during packaging [47, 63]. Such a packaging signal exists for many coronaviruses, but for SARS‐CoV‐2 has not yet been clearly identified [3, 25, 64]. Further studies on the P3 region with a focus on the impact of its secondary structure on interactions with the N‐protein would help the identification of this region as a packaging signal.

A limitation of this study is the uncertainty concerning the initial conformation of the N‐protein homodimer. In addition, the initial docking residue between the N‐protein and the RNA molecules could affect our findings. In principle, molecular dynamics simulations are the ideal tool to address this, as they allow us to capture protein conformations that are not accessible with AlphaFold Multimer (AF) [65]. However, transitions from one protein conformation to another may require longer simulation times than are available here. Indeed, intrinsically disordered proteins (IDPs) are prone to have their extended conformations be under‐sampled in molecular dynamics [66, 67, 68, 69, 70, 71]. To investigate the validity of our starting structure, we compared the radius of gyration of the predicted structures to radii of gyration as measured with small‐angle x‐ray scattering (SAXS). To this end, we used CRYSOL [72] and found radii of gyration that lie between 4 and 4.5 nm (Table S1). SAXS reports on the N‐protein find average radii of gyration of 5.2 [18] and 5.9 nm [31], which suggests that the AF predicted structures are in reasonable agreement with experimental measurements. In addition, we believe that a comparative analysis, as we have carried out, should be extensible to the whole conformational landscape. Nevertheless, there remains a risk that the conformational landscape and dynamical behavior that we have observed in our study are not representative of in vivo N‐protein structure. Sampling techniques such as Hamiltonian or temperature replica exchange molecular dynamics (H‐REMD, T‐REMD) [73, 74], metadynamics [75, 76], or targeted umbrella sampling [77] could improve sampling of the wider conformational landscape of the full‐length N‐protein homodimer, but go beyond the scope of this work.

Together, our findings indicate that phosphorylation can affect both N‐protein dimer behavior and RNA binding. They suggest that it would be worthwhile to perform a more in‐depth systematic in silico analysis on varying RNA sequences, lengths, and secondary structures in complex with full‐length N‐proteins, starting from different N‐protein structures and docking sites. Known RNA sequences from the viral genomic RNA have been shown to have unique interactions with the N‐protein [9, 10, 39, 47], which we confirm with our simulations. A general molecular framework for understanding how RNA composition influences its function is beginning to take shape [47, 59, 70, 78, 79, 80], and could be used to inspire the design of in silico screening experiments pertaining to N‐protein:RNA interactions.

## 4 Materials and Methods

### 4.1 System Preparation

The starting N‐protein homodimer structure for the molecular dynamics simulations was obtained by submitting the amino acid sequence of SARS‐CoV‐2 N‐protein (UniProt: P0DTC9) to AlphaFold Multimer [54, 55] (AF). We validated the starting structures by aligning the CTD and NTD with known crystal structures of these domains (PDB: 6WZO, 6VYO) (Figure S1). From the tested structures, we used the one that reached the lowest energy value after steepest descent energy minimization (Table S1). The protonation state of the dimer was generated with the webserver PDB2PQR [81, 82], and corresponds to physiological conditions in a cell (pH 7.4) [83]. In these conditions, the N‐protein homodimer has a net charge of +48. The NTD domain was isolated from the dimer in PyMOL and consists of residue 51–175.

For the N‐protein:RNA simulations, the initial polyU and polyA RNA structures were prepared by trimming a 1906 nt single‐stranded homopolymeric polyU, obtained from the RNA databank (PDB: 1H1K, chain I), to a 100 nt long single‐stranded polyU sequence. We subsequently docked the polyU on the N‐protein using HADDOCK [58, 84] (Figure 1D). As HADDOCK requires prior knowledge on binding sites to perform its prediction, the RNA molecule was docked on N‐protein residue 107 (Arg107), which is a known RNA binding site [52]. In the best‐scoring HADDOCK model, nucleotides from the polyU RNA molecule were further trimmed to obtain a tractable simulation system size, resulting in a final RNA length of 50 nt. The mutagenesis wizard of PyMOL was used to generate the 50 nt polyA construct, as well as both SLSL3 and SL4ext as stretched‐out structures for the NTD simulations specifically. To generate the secondary structure of the two stem‐loop regions, we used previously resolved secondary structures from [47] and the 3DRNA/DNA Web Server [85, 86, 87]. The stem‐loops were docked to protein residues Arg92 and Arg107 in the full dimer simulations and residue Arg107 in the simulations containing just the NTD. The polyU and polyA structures are docked at nucleotide 22, while SL2SL3 is docked at the TRS‐L region (nucleotides 31–36) and SL4ext at the AU‐rich sequence (nucleotides 48–64).

To generate the initial phosphorylated structures, we used PyTMs [88], a plugin of PyMOL [89]. All known phosphosites in the linker regions of both protomers were phosphorylated [34, 35] (Figure 1C). These correspond to serines in positions 176, 180, 183, 184, 186, 188, 190, 194, 197, 201, 202 and 206, at which we introduced phosphoserines ($C_{3}H_{4}{\mathrm{NO}}_{5}P^{2-}$), and threonines in positions 198 and 205 at which we introduced phosphothreonines ($C_{4}H_{6}{\mathrm{NO}}_{5}P^{2-}$). In total we introduced 28 phosphate groups, with a combined charge of −56, rendering the net charge of a phosphorylated N‐protein homodimer −8.

In total, we carried out 35 simulations across 17 unique systems (Table 1).

### 4.2 Molecular Dynamics

The parametrization of the structures for classical molecular dynamics was done with the *tleap* tool of AmberTools23 [90]. The structures were solvated in a box of water, padding the protein and RNA molecule with 15 Å, using the explicit OPC water model [91]. Na^+^ and Cl^−^ ions (Li and Merz) [92] were added to neutralize the system and reach a salt concentration of 0.15 mol dm‐3. For the protein, the Amber ff19SB force field [93] was used, with the phosaa19SB extension for phosphorylated amino acids. The OL3 force field [94] was used for the RNA molecules. For mixed protein:RNA simulations, the combination of ff19SB + OL3 + OPC appears to be the best available force field combination [95]. The parameterized files were converted from Amber to GROMACS format using ParmEd 4.1.0 [96].

All subsequent production, post‐processing and analysis was carried out using the GROMACS v2022.3 software suite [97, 98, 99, 100, 101, 102, 103] unless stated otherwise, and computationally run on the Phase 2 DelftBlue supercomputer [104], as well as the Snellius Supercomputer, hosted by SURF. The steepest descent algorithm was used to minimize the energy in the system until a maximum force of 1000 kJ mol^−1^ nm^−1^ was achieved or for a maximum of 50 000 steps. The Verlet leapfrog algorithm was used to numerically integrate the equations of motion with a time step of 2 fs. Coordinates were written every 10 ps. A cutoff of 1 nm was used for short‐range electrostatic and Van der Waals interactions. Long‐range electrostatic interactions were calculated by particle‐mesh Ewald summation [105] with a fourth‐order cubic interpolation and a grid spacing of 0.16 nm. The system was first equilibrated for 100 ps in the *NVT* ensemble using the modified Berendsen thermostat to reach an equilibrium temperature of 300 K. Then, the system was equilibrated for 100 ps in the *NpT* ensemble using the Parrinello‐Rahman [106] barostat, and produced for 1 μs with these same conditions (1 bar, 300 K). During the *NpT* equilibration and production, all hydrogen‐containing bonds were constrained using the LINCS algorithm [107].

### 4.3 Post‐Processing of the Production Output

We corrected the raw output trajectories for periodic boundary conditions (PBC) using the *whole* and *nojump* options of *gmx trjconv −pbc*, and generated trajectories saving a frame each 1 ns or each 10 ns using the skip flag of *gmx trjconv*. For visualization purposes, the trajectories obtained using the skip flag were rotationally and translationally fitted to the C‐terminal domain (CTD, residues 247–365) to gain aligned trajectories. The full trajectories were used for analyses, unless stated otherwise.

### 4.4 Analysis of the Trajectories

The shift of each protein's backbone atom in a trajectory was calculated with TrajMap [26]. TrajMap easily plots, for each residue and at each time point in a trajectory, the magnitude of shift of the backbone atoms in a heatmap, with respect to the atom's initial position. To generate the trajectory maps, the pre‐processing script from https://github.com/matkozic/TrajMap was first used to convert a 1 ns per frame trajectory into csv format. The plotting was then done with the *makemap* script, which we slightly modified to match the figure panels and labels to the N‐protein homodimer.

The intermolecular contact maps were generated with the Conan Python MD analysis tool [27]. For the interactions, we used a cutoff distance of 0.4 nm and a cutoff lifetime of 10%. We selected each atom in a residue and compared intermolecular interactions. This amounted to measurements of chain A vs. chain B for the simulations without RNA and protein vs. RNA for the simulations with RNA.

The average fluctuation in a trajectory was obtained through the root mean square fluctuation (RMSF) of the protein backbone atoms (N, $C_{\alpha }$, and C) with respect to their average position over the course of the three replicate 1 μs trajectories, both for the phosphorylated and the non‐phosphorylated simulations. The RMSF of the RNA molecules was obtained by selecting the C5 atom of each nucleotide in the analysis. Identical residue IDs (1–419) are attributed to both protomers, and thus averaged over in the RMSF, since we found no significant asymmetries in the two identical chains of each homodimer (Figure S3A,B).

To calculate the average movement of the N‐terminal domains (NTD, residues 51–174), we used the root mean squared displacement (RMSD) of the NTD with respect to its initial position in the homodimer. We also used the RMSD to calculate the movement of the RNA molecules with respect to their own initial structure. The initial RNA conformation is relatively extended for each simulation, thus a higher RMSD value corresponds to a more bent or compacted RNA molecule. The RMSD of the full protein backbone with respect to its initial structure was also obtained (Figure S3D). We measured the distance between NTD and the CTD by selecting the center of mass of the NTD and the closest residue of the CTD to this center of mass at t=0. This way, movement of the NTD along the radial profile of the CTD could be captured (Figure S4B).

The number of hydrogen bonds was obtained using a cutoff distance of 0.35 Å and a cutoff angle of 30°. We compute the probability density by binning the number at each time point, resulting in either 300 000 counts for the simulations performed in triplicate or 100 000 counts for the 1 μs simulations.

The commands used to generate the root mean square displacement, root mean square fluctuation, radius of gyration, NTD‐CTD distances, linker and RNA end‐to‐end distances, NTD‐CTD‐NTD angle, and the number of hydrogen bonds were, respectively, (*gmx*) *rms*, *rmsf*, *gyrate*, *distance*, *polystat*, *gangle*, and *hbond*.

The amount of salt bridges in a simulation was extracted using the *saltbridges* plugin of Visual Molecular Dynamics (VMD) [108] and the trajectories with 1 ns between each frame. Herein, a salt bridge is taken to be an oxygen‐nitrogen atom pair that lies within a cutoff‐distance of 4 Å. The plugin takes into account all acidic (Asp, Glu) and basic (Arg, His, Lys) residues in the simulation. We compute the probability density by obtaining the number of salt bridges at a set interval of 1 ns using the complete 3 μs of the non‐phosphorylated and phosphorylated trajectories.

To obtain the binding affinities we used the Molecular Mechanics energies with Generalized Born and Surface Area continuum solvation method (MM/GBSA), as described in [57, 109]. Here, the free energy is calculated using Equation (1). The bonded, electrostatic and Van der Waals interaction energy terms are calculated via molecular mechanics, the polar solvation term $G_{\mathrm{pol}}$ by employing generalized Born, and the non‐polar term $G_{\mathrm{np}}$ from a linear relation to the solvent accessible surface area.

(1) $$G=E_{\mathrm{bnd}}+E_{\mathrm{el}}+E_{\mathrm{vdW}}+G_{\mathrm{pol}}+G_{\mathrm{np}}-\mathrm{TS}$$

(2) $$\Delta G_{\text{bind}}=G_{\mathrm{AB}}-G_{A}-G_{B}$$

The entropy term is often omitted as it does not improve the final result, while being the most computationally demanding term. It is therefore also omitted in our calculations. Because of this, the obtained values are most reliable in their relative values, and we thus evaluate the sign of the ΔΔG and of the free energy contributions, instead of their absolute values. The sum of the energy contributions is calculated for each protomer and RNA molecule in the simulations, as well as their combined complexes, yielding a total Gibbs energy of binding through Equation (2).

We calculated the binding affinities using the last 500 ns of each trajectory with 1 ns between each frame. ΔΔ *G* is subsequently obtained by taking the difference between $\Delta G_{\mathrm{Ph}}$ and $\Delta G_{\text{nonPh}}$ ($\Delta \Delta G=\Delta G_{\mathrm{Ph}}-\Delta G_{\text{nonPh}}$) where a positive value indicates a destabilizing influence of phosphorylation, and vice versa for a negative value. To compare the different RNA conformations we took the difference between $\Delta G_{\text{folded}}$ and $\Delta G_{\text{stretched}}$ ($\Delta \Delta G=\Delta G_{\text{folded}}-\Delta G_{\text{stretched}}$).

## Author Contributions

**Stefan Loonen:** conceptualization, investigation, writing – original draft, writing – review and editing, visualization, validation, methodology, software, formal analysis, resources, data curation, supervision. **Lina van Steenis:** conceptualization, methodology, software, data curation, investigation, validation, formal analysis, visualization, resources, writing – original draft, writing – review and editing. **Marianne Bauer:** conceptualization, investigation, funding acquisition, writing – review and editing, validation, methodology, project administration, resources, supervision, visualization. **Nikolina Šoštarić:** conceptualization, investigation, writing – review and editing, visualization, validation, methodology, resources, supervision.

## Conflicts of Interest

The authors declare no conflicts of interest.

## Peer Review

The peer review history for this article is available at https://www.webofscience.com/api/gateway/wos/peer‐review/10.1002/prot.26842.

## Supporting information


**Data S1.** Supporting Information.

## Data Availability

The following files are available: (1) the raw trajectories (available on request), (2) the PBC‐corrected full trajectories (10 ps between each frame; available on request), (3) the aligned and skipped trajectories (1 and 10 ns between each frame, available on DOI: 10.4121/f2eeef37‐4e13‐4462‐bf89‐c3afb709c098).
