# Supplementary material for: Phosphorylation Changes SARS‐CoV‐2 Nucleocapsid Protein's Structural Dynamics and Its Interaction With RNA
Source: Proteins. 2025 May 15;93(10):1701–16. doi: 10.1002/prot.26842 (PMC12433262; doi:10.1002/prot.26842)
Supplement: Supplementary file 1 — Data S1. Supporting Information. [file PROT-93-1701-s001.pdf]

Supplementary information:
Phosphorylation changes SARS-CoV-2 nucleocapsid protein's structural dynamics and its interaction with RNA

Running title: Structural dynamics of SARS-CoV-2 N-protein

Stefan Loonen,[1] **Lina van Steenis**,[1] **Marianne Bauer**,[1,2,*] **Nikolina Šoštarić** [1,2,*]
[1] Department of Bionanoscience, Kavli Institute of Nanoscience Delft, Delft University of Technology, Van der Maasweg 9, 2629 HZ Delft, The Netherlands
[2] Lead Contact
[*] Correspondence: m.s.bauer@tudelft.nl n.sostaric@tudelft.nl

Table S1: The potential energy after steepest descent energy optimization, RMSD of the alignment of the CTD and NTD domains with the respective crystal structures and the radius of the hydration shell, as measured with CRYSOL[1], are shown for the top five predicted AlphaFold Multimer[2] structures. Model 2 and 5 achieve the lowest potential energy. All structures have alignments with RMSD values below 1 Å and their radius of gyration is measured to be between 4.1 and 4.3 nm.

Structure	Potential energy after minimization (kJ/mol)	Alignment RMSD CTD (Å)	Alignment RMSD NTD (Å)	Shell Rg CRYSOL (nm)
Model 1	-4.8e+06	0.38	0.47	4.1
Model 2	-7.9e+06	0.39	0.56	4.3
Model 3	-6.0e+06	0.40	0.64	4.1
Model 4	-6.7e+06	0.41	0.68	4.3
Model 5	-7.9e+06	0.38	0.60	4.2

Table S2: Overview of the measured salt bridges in the different replicates of the trajectories of non-phosphorylated and phosphorylated N-protein, as measured by the VMD[3] plugin *Salt-bridges*. The percentages indicate the proportion of the trajectory that the particular salt bridge was within the cut-off distance of 4 Å. For this analysis the trajectories that have a spacing of 1 ns between each frame were used. In the residue labeling the counting does not reset for residues in the second protomer, thus 1-419 corresponds to protomer A and 420-838 to protomer B.

Simulation state	Salt bridge	Occupation time
Non-phosphorylated replicate 1	Asp 501 - Arg 455	77.62 %
	Asp 547 - Arg 508	69.73 %
	Asp 644 - Arg 628	51.85 %
	Asp 82 - Arg 36	67.53 %
	Asp 82 - Lys 143	55.74 %
	Glu 118 - Lys 127	83.72 %
	Glu 174 - Arg 107	65.83 %
	Glu 323 - Arg 596	57.44 %
	Glu 537 - Lys 546	74.83 %
	Glu 593 - Arg 526	63.64 %
Non-phosphorylated replicate 2	Asp 348 - Arg 185	53.15 %
	Asp 358 - Arg 738	82.82 %
	Asp 547 - Arg 508	60.54 %
	Glu 118 - Lys 127	75.12 %
	Glu 174 - Arg 107	72.33 %
	Glu 537 - Lys 546	66.83 %
	Glu 593 - Arg 526	86.11 %
	Glu 709 - Arg 712	64.34 %
	Glu 742 - Arg 95	57.44 %
Non-phosphorylated replicate 3	Asp 225 - Arg 209	53.55 %
	Asp 297 - Lys 248	63.64 %
	Asp 644 - Lys 668	64.74 %
	Glu 118 - Lys 127	71.03 %
	Glu 174 - Arg 107	80.82 %
	Glu 231 - Lys 676	52.45 %
	Glu 253 - Lys 256	54.55 %
	Glu 537 - Lys 546	75.32 %
	Glu 709 - Arg 712	52.55 %
Phosphorylated replicate 1	Asp 716 - Lys 652	81.02 %
	Glu 118 - Lys 127	77.92 %
	Glu 290 - Arg 293	54.75 %
	Glu 537 - Lys 546	71.63 %
	Glu 699 - Arg 107	62.04 %
Phosphorylated replicate 2	Asp 297 - Lys 233	65.23 %
	Asp 402 - Arg 276	58.74 %
	Glu 118 - Lys 127	77.82 %
	Glu 537 - Lys 546	79.02 %
Phosphorylated replicate 3	Asp 3 - Arg 32	75.52 %
	Glu 118 - Lys 127	82.72 %
	Glu 537 - Lys 546	81.42 %
	Glu 709 - Arg 712	74.63 %
	Glu 786 - Arg 459	96.00 %

Table S3: Results of the Molecular Mechanics energies with Generalized Born and Surface Area continuum solvation method analysis. The last half of the trajectories was used to calculate the free energy of binding. In the simulations without RNA, the free energy of binding between the two respective protomers in the dimer was calculated. In the simulations containing RNA, the free energy of binding between the N-protein and the respective RNA molecules was calculated. Only the sign of the difference between non-phosphorylated and phosphorylated simulations is informative, as we have not calculated the entropic term ($\Delta\Delta G = \Delta G_{\mathrm{Ph}} - \Delta G_{\mathrm{nonPh}}$). A positive value of the $\Delta\Delta G$ indicates a destabilizing influence of phosphorylation, and vice versa for a negative value.

**Replicate**	**PTM state**	**RNA presence**	**Average ΔG (kcal/mol)**	**Std. Dev.**
1	non-phosphorylated	no RNA	-382.6	24.3
2	non-phosphorylated	no RNA	-343.9	17.0
3	non-phosphorylated	no RNA	-297.8	12.0
1	phosphorylated	no RNA	-323.2	20.3
2	phosphorylated	no RNA	-291.6	15.0
3	phosphorylated	no RNA	-312.7	18.7
N.A.	non-phosphorylated	polyU	-589.1	41.2
N.A.	phosphorylated	polyU	-289.2	29.9
N.A.	non-phosphorylated	polyA	-224.0	39.1
N.A.	phosphorylated	polyA	-91.0	31.2
N.A.	non-phosphorylated	SL2SL3	-360.5	27.6
N.A.	phosphorylated	SL2SL3	-98.3	20.9
N.A.	non-phosphorylated	SL4ext	-385.5	21.0
N.A.	phosphorylated	SL4ext	-71.0	27.3
1	NTD	polyU	-88.35	14.95
2	NTD	polyU	-139.17	18.27
3	NTD	polyU	-37.35	9.24
1	NTD	polyA	-38.45	5.96
2	NTD	polyA	-45.49	5.03
3	NTD	polyA	-61.10	13.17
1	NTD	SL2SL3 folded	-91.59	12.64
2	NTD	SL2SL3 folded	102.48	13.50
3	NTD	SL2SL3 folded	-52.60	18.03
1	NTD	SL2SL3 stretched	-86.39	17.03
2	NTD	SL2SL3 stretched	-69.97	21.23
3	NTD	SL2SL3 stretched	-87.15	19.02
1	NTD	SL4ext folded	-113.35	13.09
2	NTD	SL4ext folded	-136.11	13.89
3	NTD	SL4ext folded	-137.25	17.43
1	NTD	SL4ext stretched	-10.64	1.97
2	NTD	SL4ext stretched	-80.10	12.90
3	NTD	SL4ext stretched	-65.33	8.91

Table S4: The RNA sequences used in simulations. The polyU and polyA are each 50 nt long, the SL2SL3 41 nt and SL4ext 67 nt.

RNA molecule	RNA sequence
polyU	5’ UUUUUUUUUUUUUUUUUUUUUUUUUUUUUUUUUUUUUUUUUUUUUUUUUU 3’
polyA	5’ AAAAAAAAAAAAAAAAAAAAAAAAAAAAAAAAAAAAAAAAAAAAAAAAAA 3’
SL2SL3	5’ CUUUCGAUCUCUUGUAGAUCUGUUCUCUAAACGAACUUUAA 3’
SL4ext	5’ UCUGUGUGGCUGUCACUCGGCUGCAUGCUUAGUGCACUCACGCAGUAUAAUUAAUAACUAAUUACUG 3’

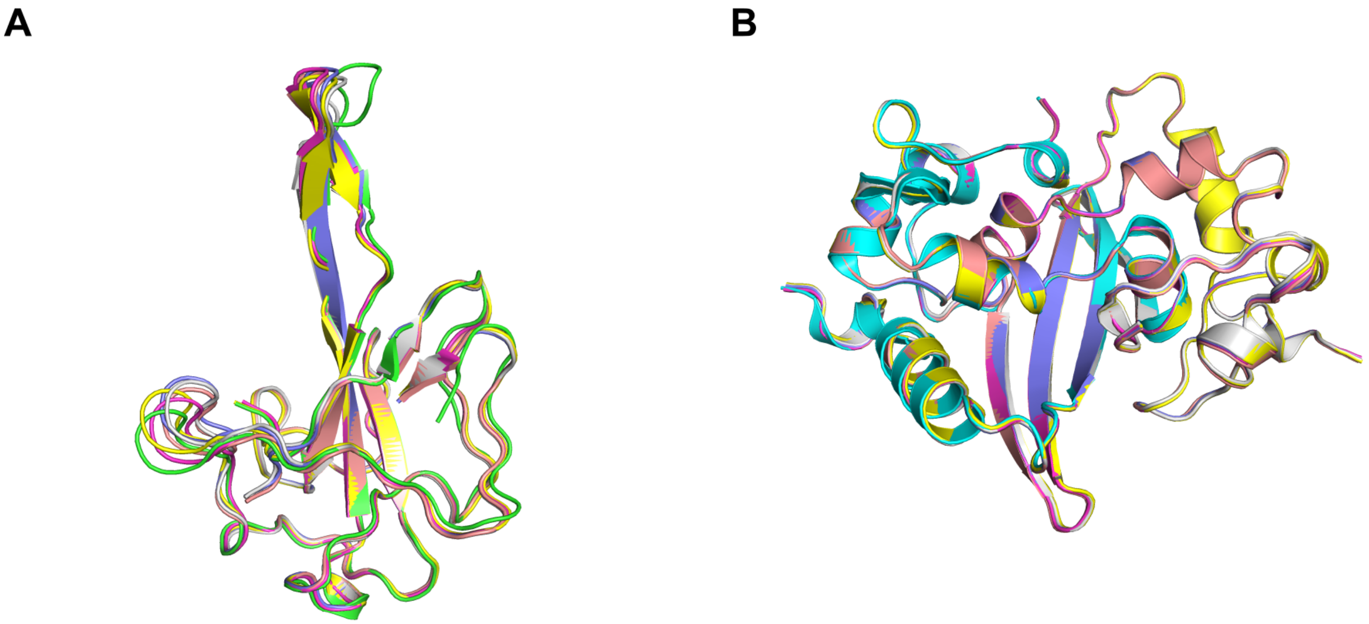

Figure S1: Top five predicted AlphaFold structures aligned to the crystal structures of **A:** the NTD (PDB: 6VYO) and **B:** the CTD (PDB: 6WZO) domains of the N-protein. The structures are shown in cartoon mode with varying colors (chosen at random). The differences between the full-length AlphaFold structures lie in the unstructured regions of the respective domains.

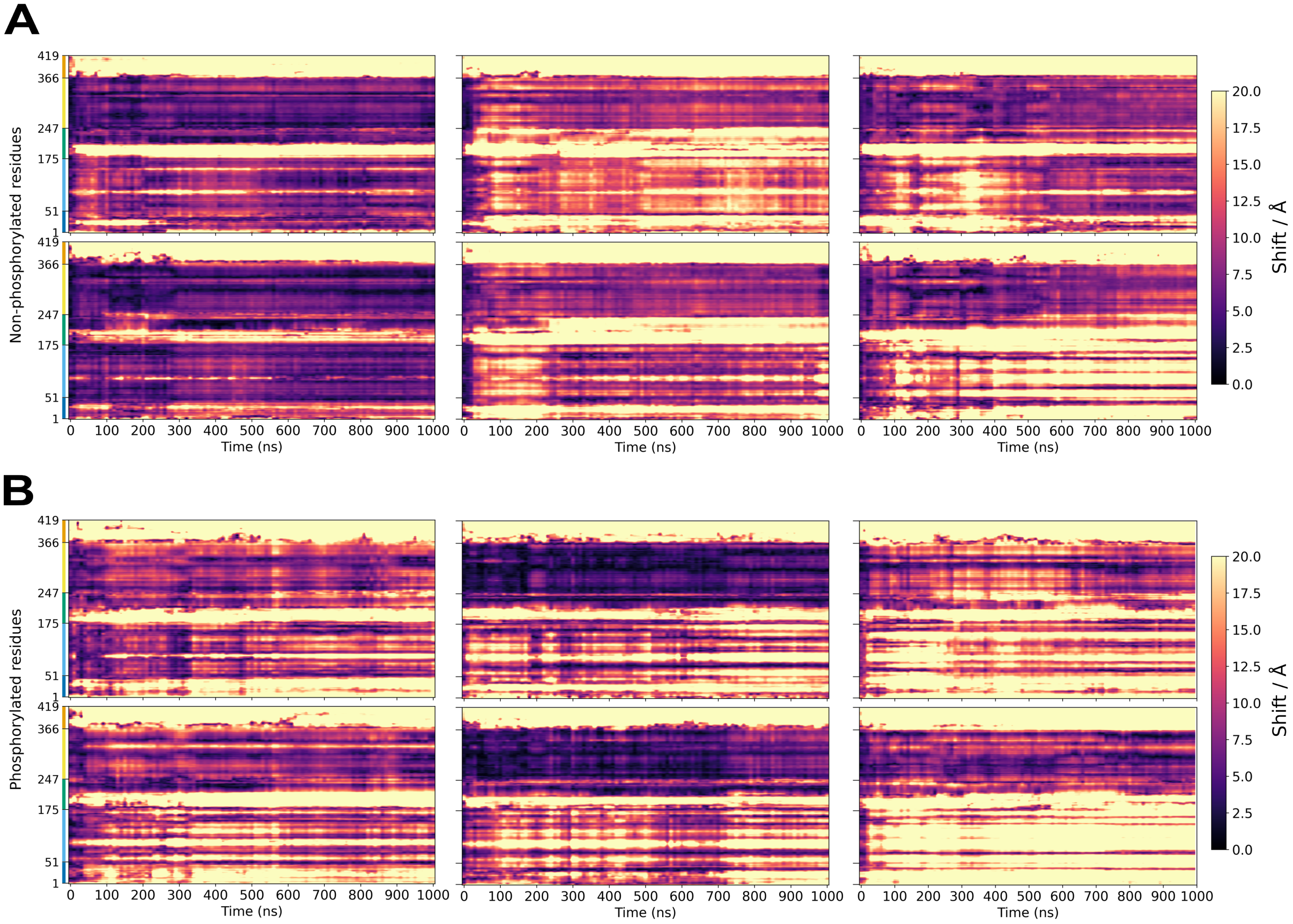

Figure S2: Trajectory maps generated using the TrajMap Python suite[4]. **A:** The top row shows three replicates of the non-phosphorylated N-protein molecular dynamics simulations and **B:** the bottom row three replicates of the phosphorylated N-protein. The shift of the backbone atoms is calculated, for each residue and at each time point in a trajectory, with respect to the atom's initial position. The shading of a cell indicates the magnitude of the shift, with a dark color (black) corresponding to a low value and a light color (yellow) to a high value. For each simulation we produce two heatmaps, corresponding to both protomers that constitute a dimer, which in the panel are stacked on top of one another. In the resulting figures stretches of yellow shading indicate a high deviation from the initial reference structure.

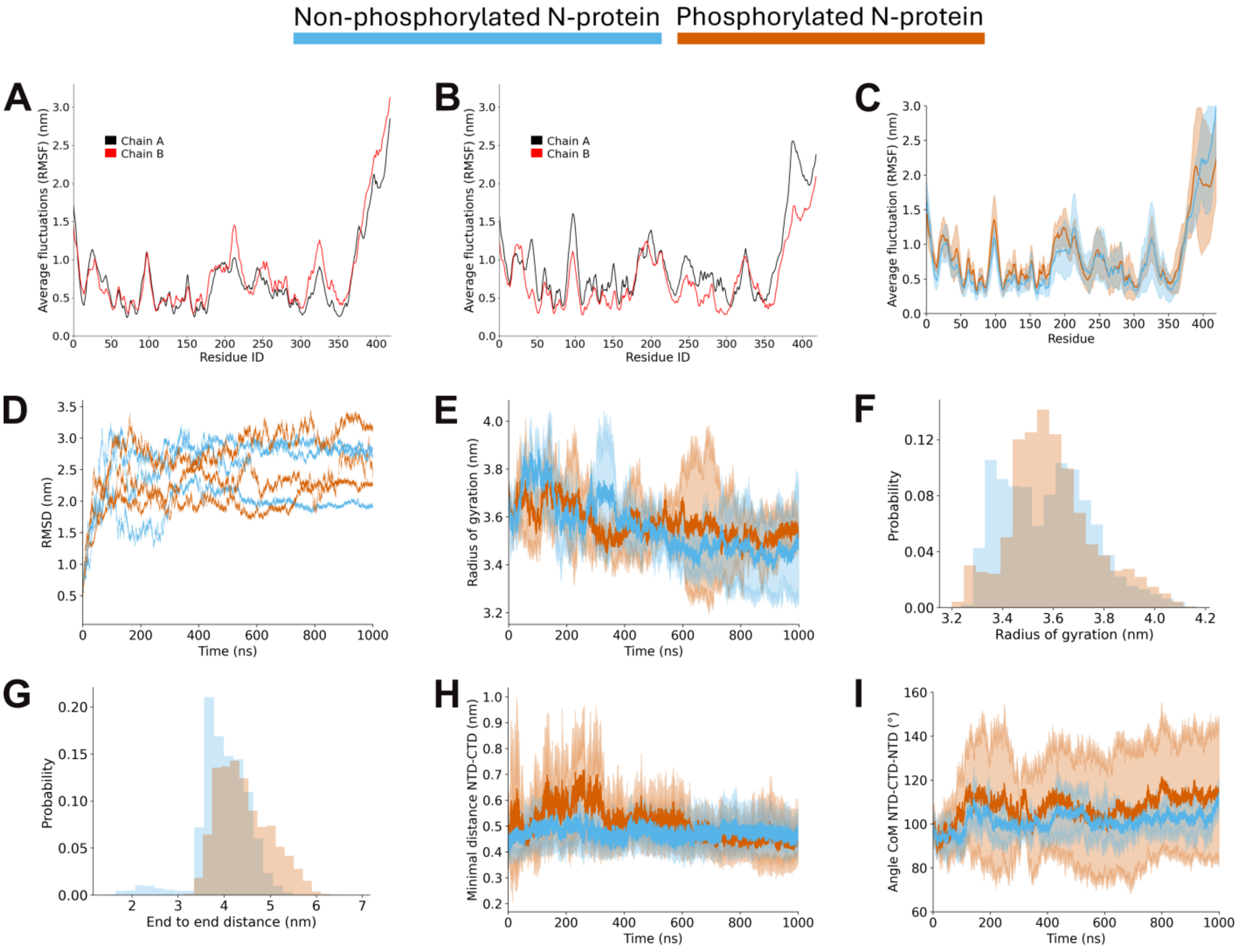

Figure S3: A: The root mean square fluctuations (RMSF) of chain A (black) and chain B (red) is shown for non-phosphorylated and **B:** phosphorylated N-protein, where the average is taken for three 1 μs replicates. **C:** The mean RMSF and standard deviation for both chains is shown. **D:** The root mean square deviation (RMSD) of the protein backbone atoms with respect to their initial position is shown for all three replicates of both the non-phosphorylated and phosphorylated N-protein. **E:** The mean radius of gyration of the protein for non-phosphorylated (blue) and phosphorylated (red) N-protein. **F:** Histogram of binned values of the radius of gyration of non-phosphorylated and phosphorylated N-protein at each time-step. For a comparison with experimental values, see [5]. **G:** Histogram of binned values of the end-to-end distances of each protomer in a dimer across trajectories for non-phosphorylated and phosphorylated N-protein. For a comparison with experimental values, see [5]. **H:** The minimal distance between the NTD and CTD is shown, where the average and standard deviation are calculated from three 1 μs replicates. A schematic representation of this distances is shown in Figure S4A. **I:** The angle that is spanned by the center of mass of either NTD of the two protomers and the center of mass of the combined CTD is shown for the non-phosphorylated and phosphorylated N-protein.

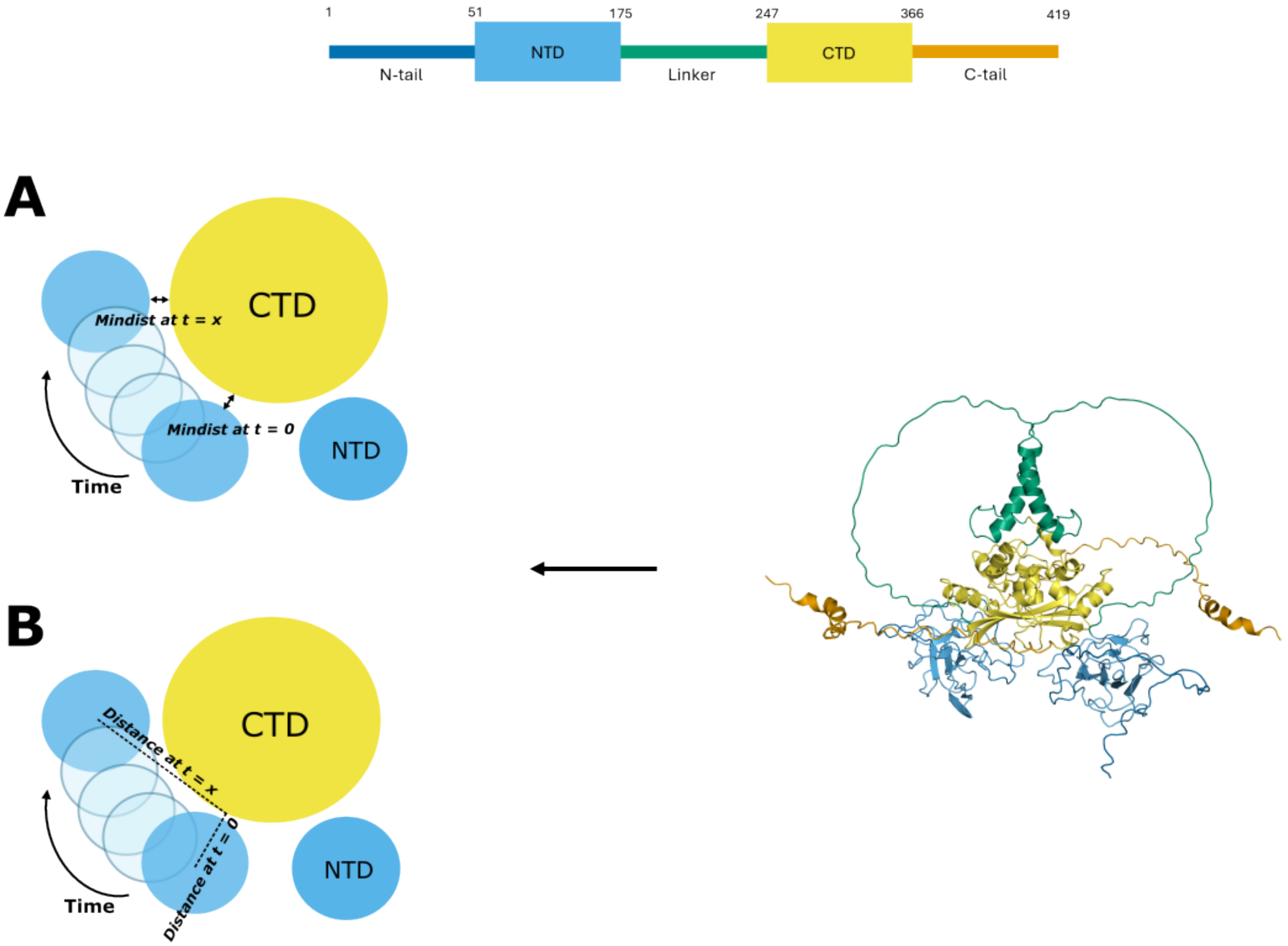

Figure S4: A: Schematic representation of the minimal distance between the NTD and the CTD, used to analyse the different trajectories of the N-protein. **B:** Schematic representation of the distance between the centre of mass of the NTD and the initial closest residue of the CTD, used to analyse the different trajectories of the N-protein. The original AlphaFold structure is shown in cartoon representation and colored according to the legend.

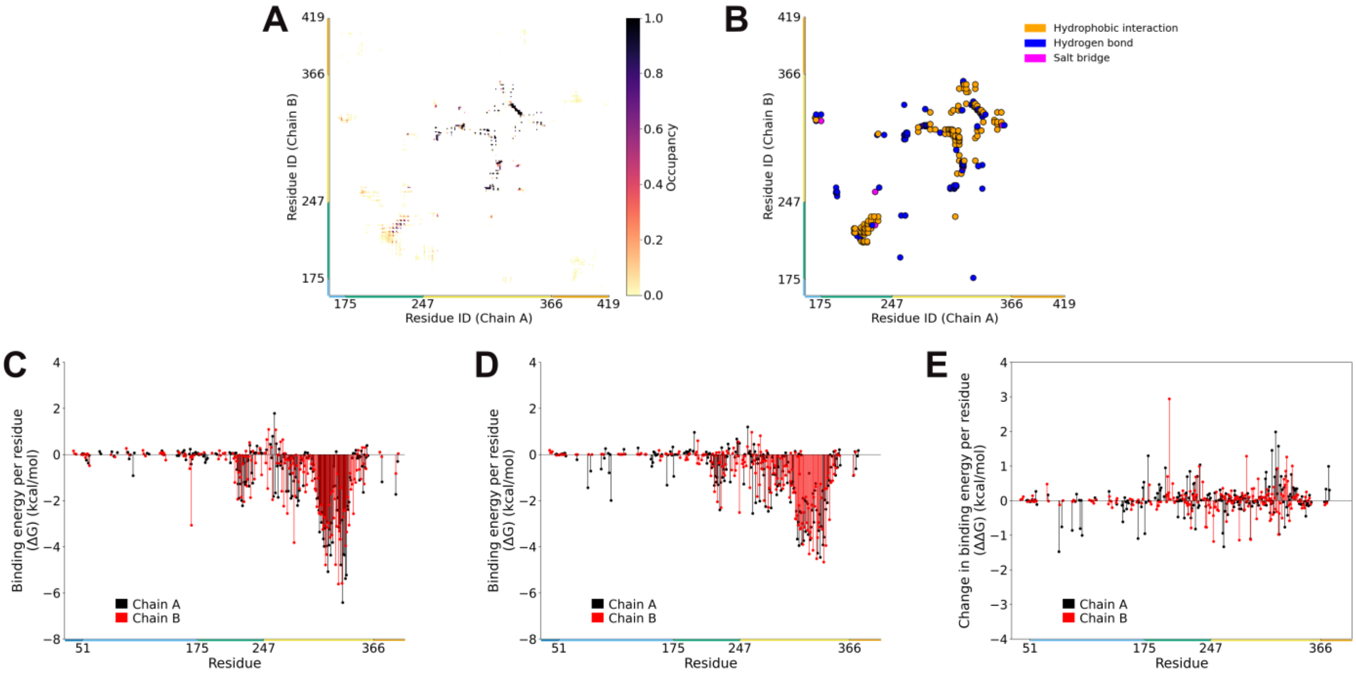

Figure S5: A: Intermolecular contact map generated using the Conan Python suite[6]. Contacts are shown between chain A and chain B of the phosphorylated N-protein homodimer. For an interaction we take a cutoff distance of 0.4 nm and a cutoff occupancy of 10%. We plot the average over three replicates and do not show the first 160 residues to highlight the contacts that mediate the dimerization interaction. The shading corresponds to the average occupancy of a particular contact. We recognize residues in the CTD which are known to mediate dimerization, and thus are expected to be in contact. **B:** Interaction types of the contact plotted in **A**. To generate the type of an interaction the protein pdb was parsed into Conan which, based on the residue types, assigns an interaction type. The dimerization is mainly mediated through hydrophobic interactions and hydrogen bonds. **C:** Residue specific contributions to the free energy of binding between chain A (black) and chain B (red) are shown for non-phosphorylated N-protein and **D:** phosphorylated N-protein. **E:** Difference between the per residue ΔG_{Ph} and $\Delta G_{\mathrm{nonPh}}$ is shown. A non-zero difference indicates that residues have a different contribution to the binding energy between the non-phosphorylated and phosphorylated simulations. A positive value corresponds to a destabilizing effect of phosphorylation on the contribution of the residue to binding and a negative value a stabilizing effect.

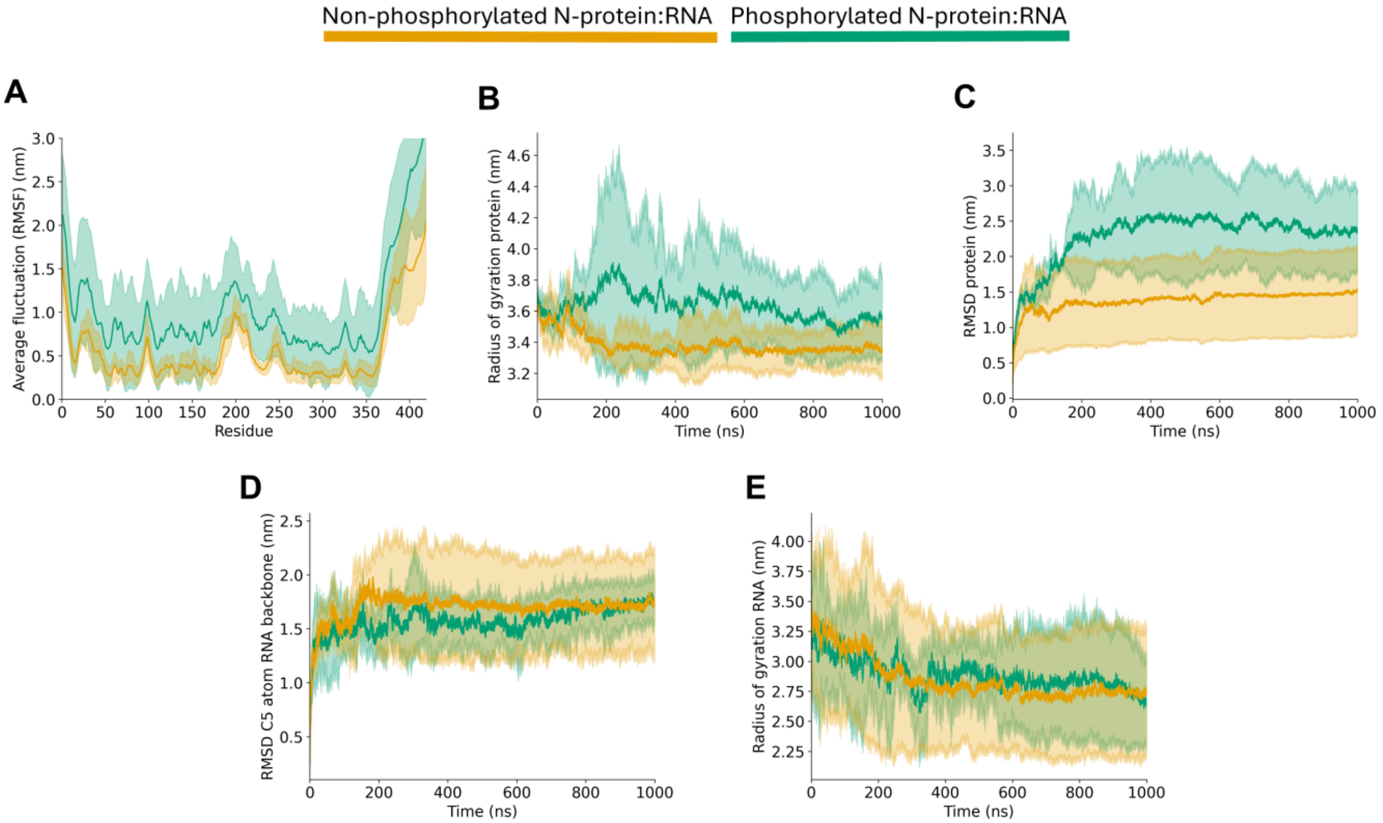

Figure S6: The mean and standard deviation of four simulations with varying RNA molecules are shown for several measures. Non-phosphorylated N-protein is plotted in yellow and phosphorylated N-protein in green. **A:** The RMSF of the protein backbone atoms. **B:** The radius of gyration of the protein. **C:** The root mean square deviation of the protein with respect to its initial structure. **D:** The root mean square deviation of the C5 atom of the RNA backbone. **E:** The radius of gyration of the RNA molecules.

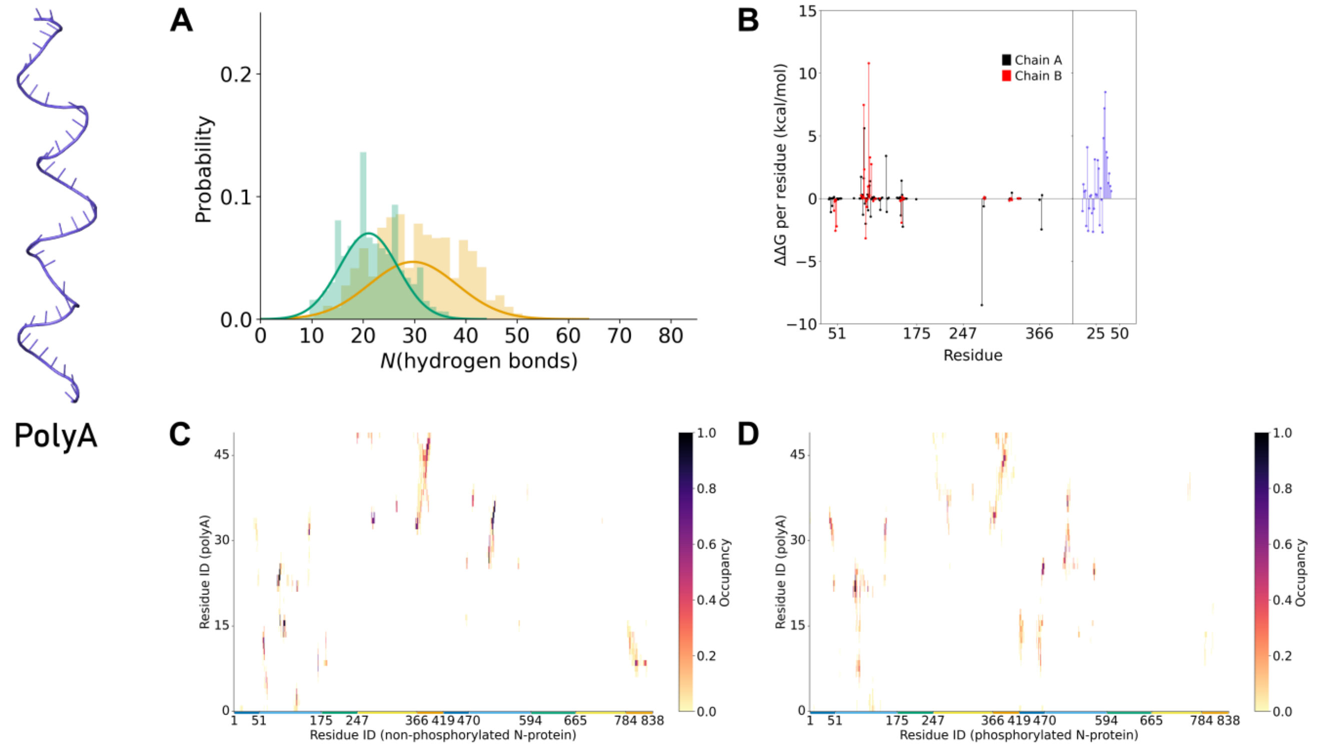

Figure S7: Residue specific measures of the polyA molecule. **A:** Probability density function of the number of hydrogen bonds formed between RNA and the N-protein. The number of counts in the histograms is $N = 100,000$. **B:** The change in binding energy between the N-protein and the polyA as a result of phosphorylation. There are two subplots; the first shows the residues of the protein, and the second shows the residues of the polyA. The two protomers are colored in black and red and plotted in the same graph. The $\Delta\Delta G$ is calculated by subtracting the non-phosphorylated ΔG from the phosphorylated ΔG ($\Delta\Delta G = \Delta G_{\text{Ph}} - \Delta G_{\text{nonPh}}$) . **C:** The intermolecular contact map, generated with Conan, between polyA and the non-phosphorylated N-protein. For an interaction we take a cutoff distance of 0.4 nm and a cutoff occupancy of 10%. **D:** The intermolecular contact map with phosphorylated N-protein and the RNA molecule.

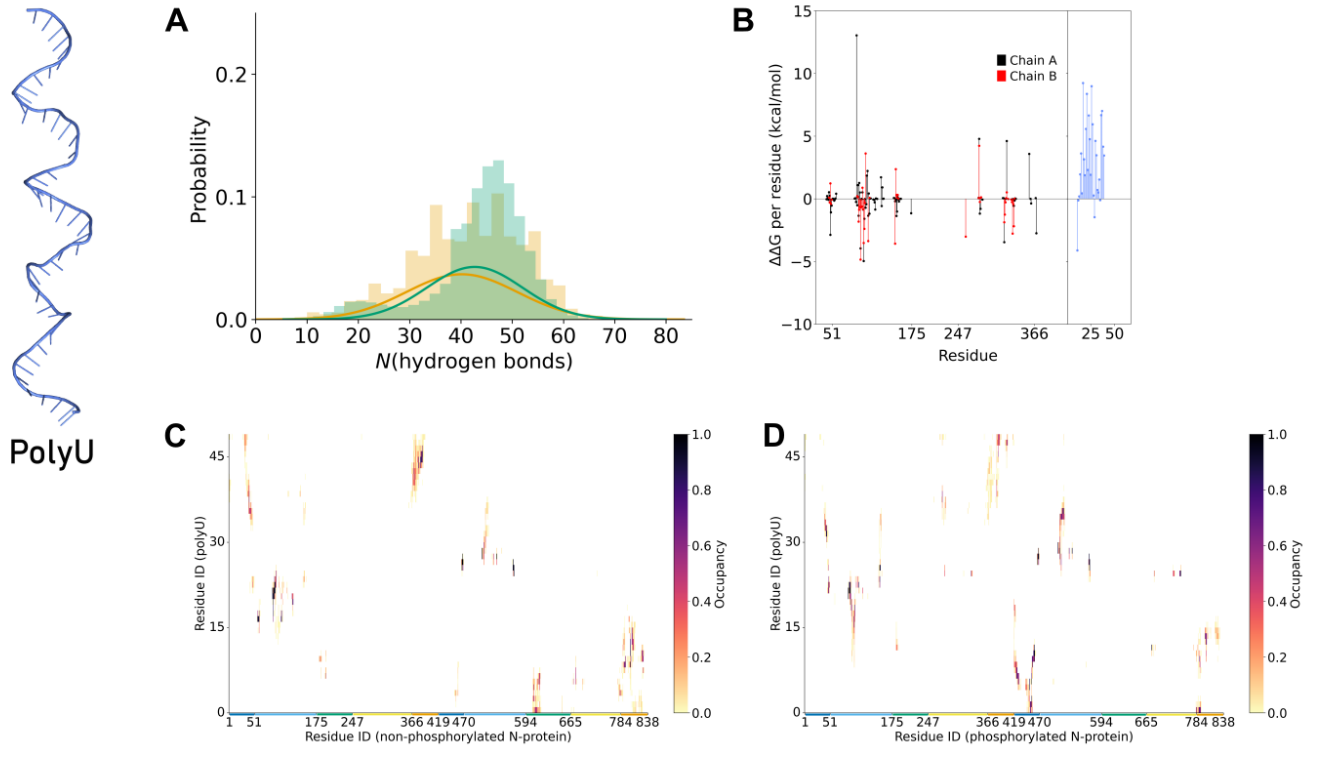

Figure S8: Residue specific measures of the polyU molecule. **A:** Probability density function of the number of hydrogen bonds formed between RNA and the N-protein. The number of counts in the histograms is $N = 100,000$. **B:** The change in binding energy between the N-protein and the polyU as a result of phosphorylation. There are two subplots; the first shows the residues of the protein, and the second shows the residues of the polyU. The two protomers are colored in black and red and plotted in the same graph. The $\Delta\Delta G$ is calculated by subtracting the non-phosphorylated ΔG from the phosphorylated ΔG ($\Delta\Delta G = \Delta G_{\text{Ph}} - \Delta G_{\text{nonPh}}$) . **C:** The intermolecular contact map, generated with Conan, between polyU and the non-phosphorylated N-protein. For an interaction we take a cutoff distance of 0.4 nm and a cutoff occupancy of 10%. **D:** The intermolecular contact map with phosphorylated N-protein and the RNA molecule.

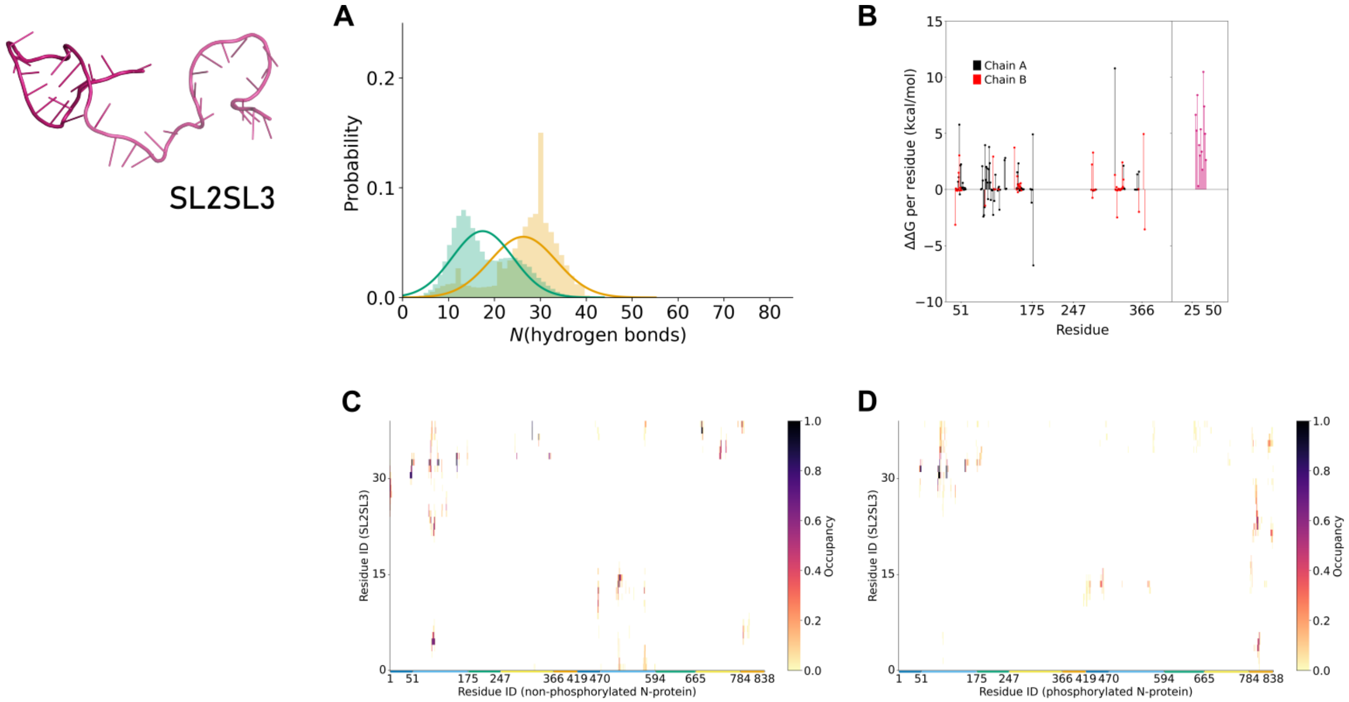

Figure S9: Residue specific measures of the SL2SL3 molecule. **A:** Probability density function of the number of hydrogen bonds formed between RNA and the N-protein. The number of counts in the histograms is $N = 100,000$. **B:** The change in binding energy between the N-protein and the SL2SL3 as a result of phosphorylation. There are two subplots; the first shows the residues of the protein, and the second shows the residues of the SL2SL3. The two protomers are colored in black and red and plotted in the same graph. The $\Delta\Delta G$ is calculated by subtracting the non-phosphorylated ΔG from the phosphorylated ΔG ($\Delta\Delta G = \Delta G_{\text{Ph}} - \Delta G_{\text{nonPh}}$) . **C:** The intermolecular contact map, generated with Conan, between SL2SL3 and the non-phosphorylated N-protein. For an interaction we take a cutoff distance of 0.4 nm and a cutoff occupancy of 10%. **D:** The intermolecular contact map with phosphorylated N-protein and the RNA molecule.

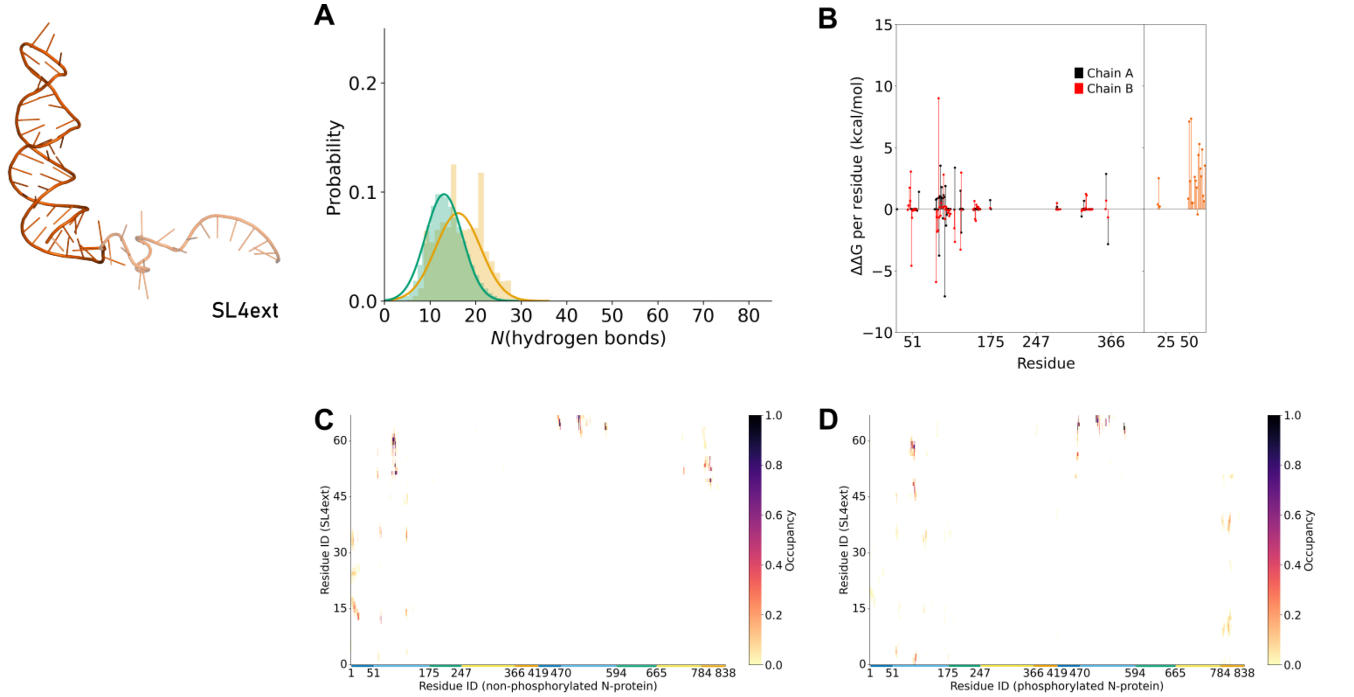

Figure S10: Residue specific measures of the SL4ext molecule. **A:** Probability density function of the number of hydrogen bonds formed between RNA and the N-protein. The number of counts in the histograms is $N = 100,000$. **B:** The change in binding energy between the N-protein and the SL4ext as a result of phosphorylation. There are two subplots; the first shows the residues of the protein, and the second shows the residues of the SL4ext. The two protomers are colored in black and red and plotted in the same graph. The $\Delta\Delta G$ is calculated by subtracting the non-phosphorylated ΔG from the phosphorylated ΔG ($\Delta\Delta G = \Delta G_{\text{Ph}} - \Delta G_{\text{nonPh}}$) . **C:** The intermolecular contact map, generated with Conan, between SL4ext and the non-phosphorylated N-protein. For an interaction we take a cutoff distance of 0.4 nm and a cutoff occupancy of 10%. **D:** The intermolecular contact map with phosphorylated N-protein and the RNA molecule.

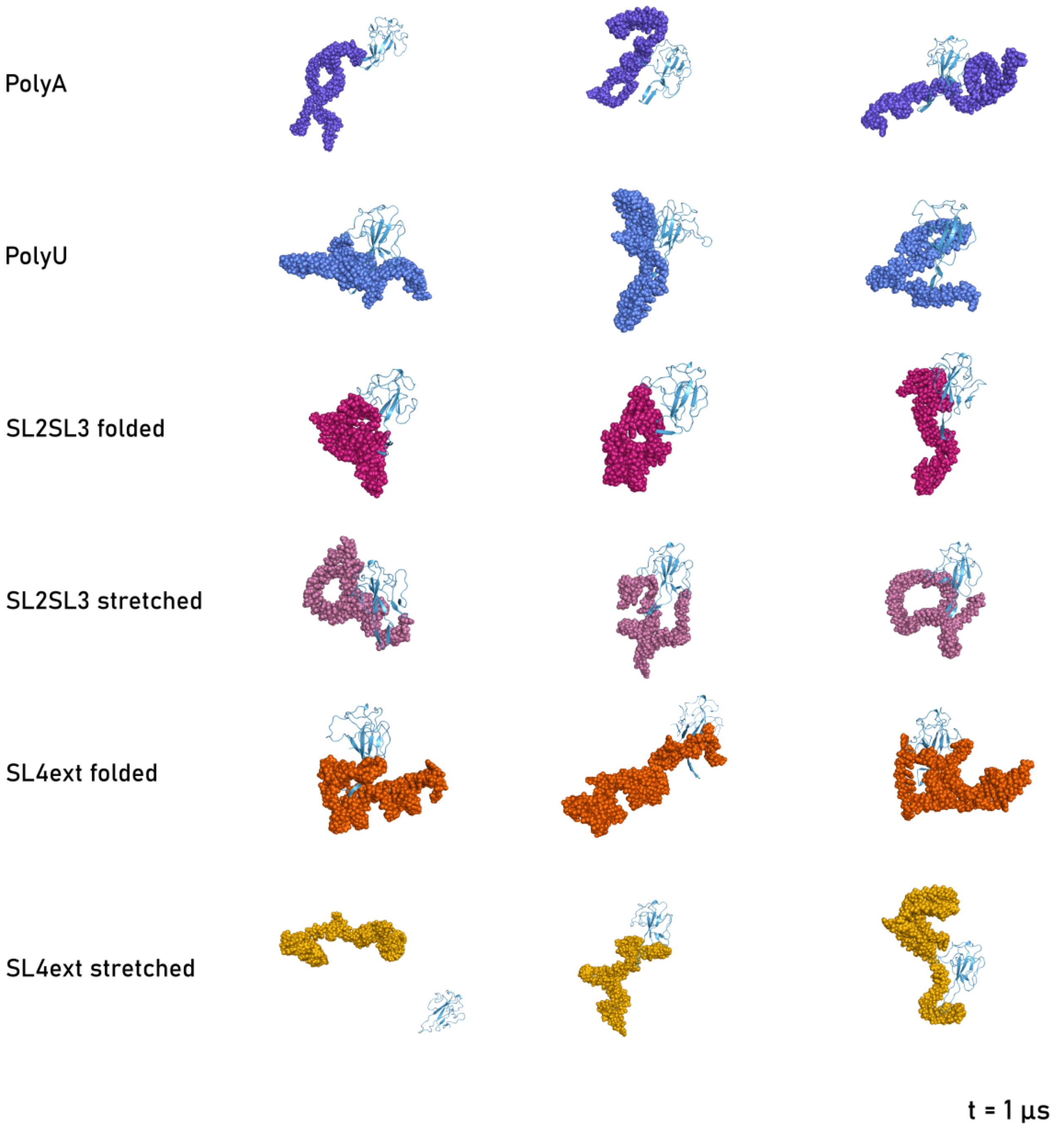

Figure S11: The endpoints of each simulation of the NTD in complex with the respective RNA type are shown. The RNA molecules are colored according to the color code introduced in Table 1 and shown using the spheres representation of PyMOL. The protein is shown in cartoon representation, colored in blue and oriented with the beta coil pointing to the bottom left.

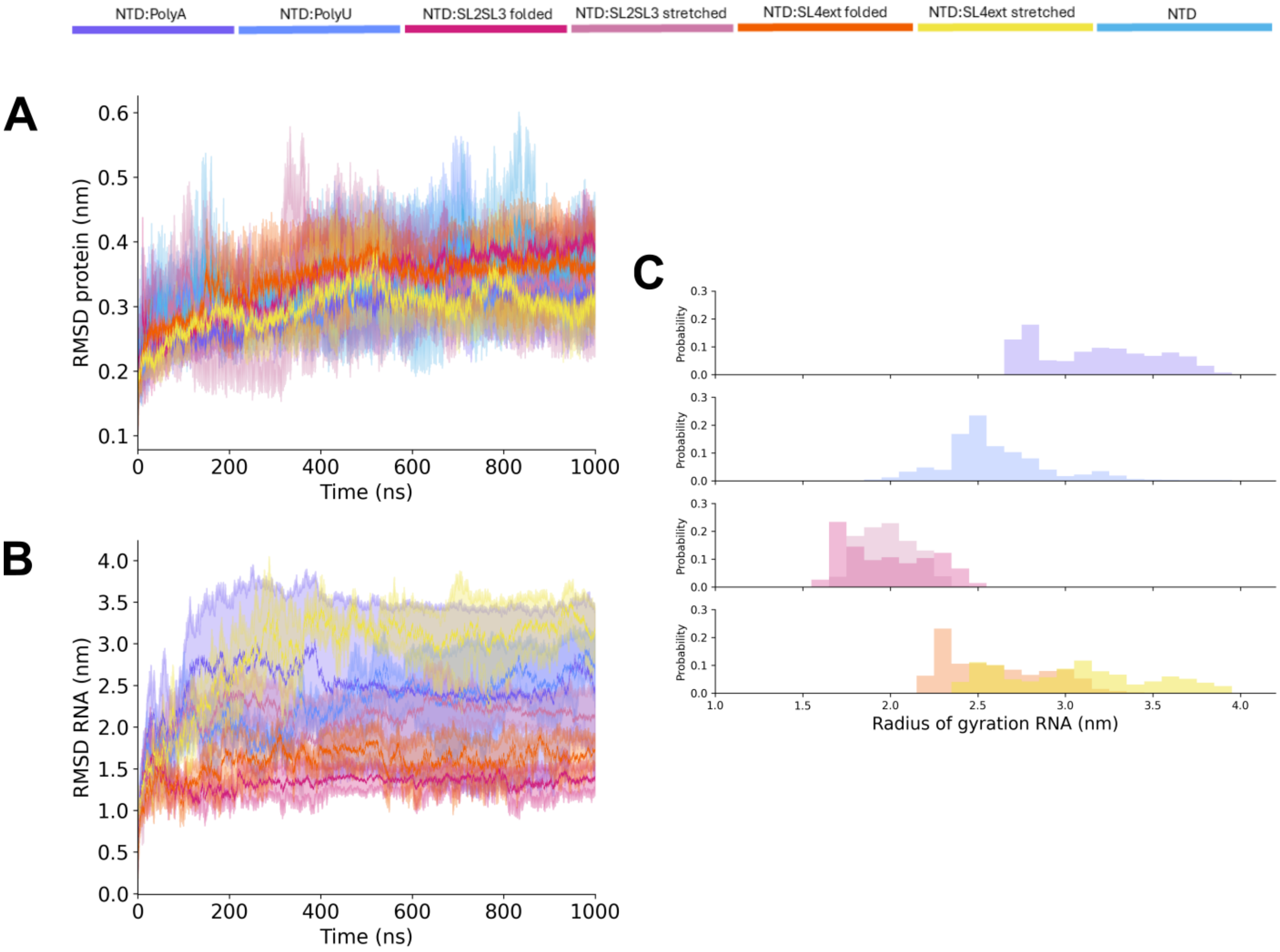

Figure S12: A: The RMSD of the NTD. The mean and standard deviation are calculated from the three replicates for each simulation. **B:** The mean RMSD of the RNA molecules only. Mean and standard deviation are calculated over the three replicates. **C:** Distribution of the radius of gyration of the simulated systems in the last 500 ns of each trajectory. Each histogram thus contains $N = 3 * 50,000 = 150,000$ counts.

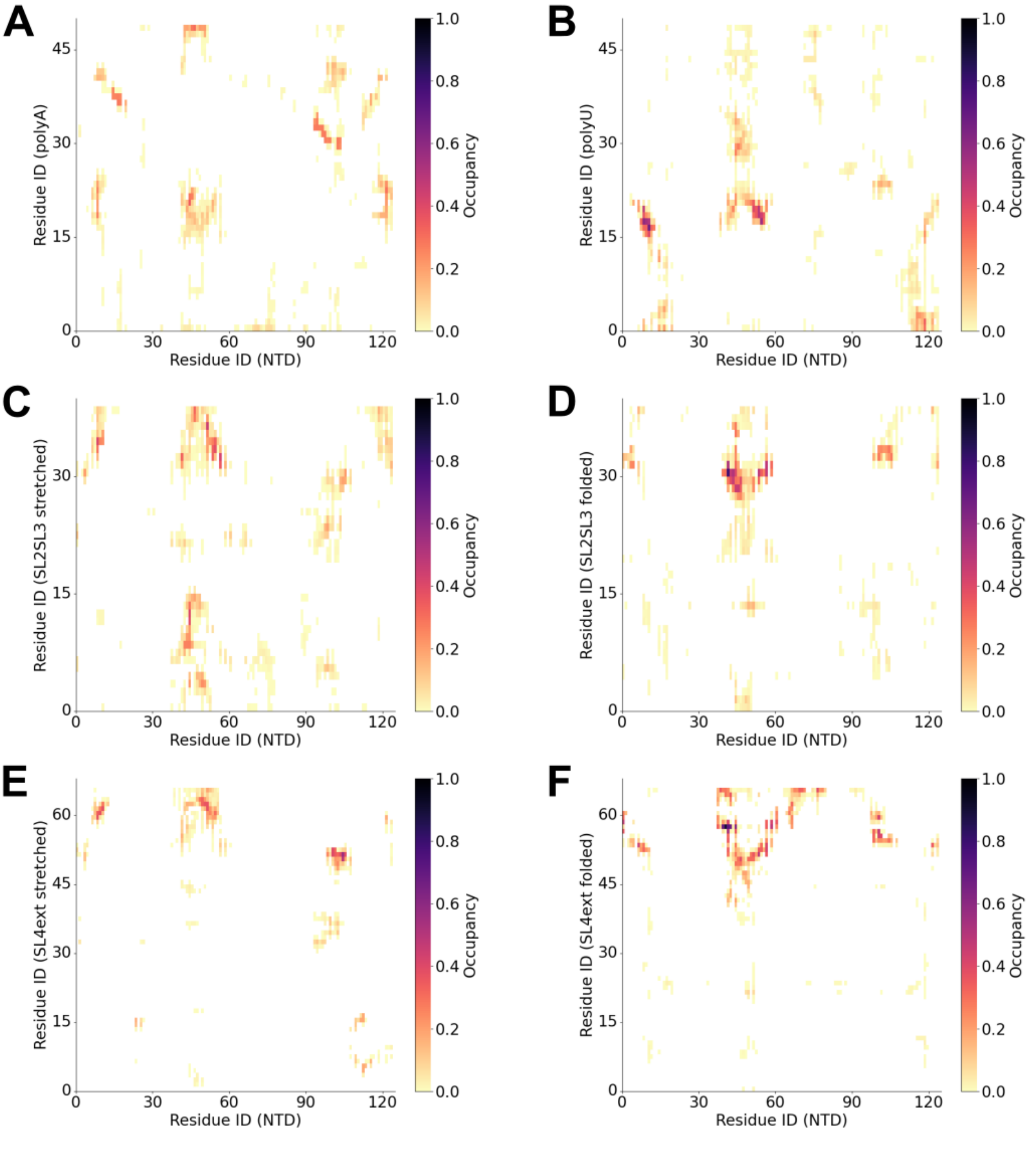

Figure S13: Intermolecular contact maps for each NTD:RNA system. The contact maps were generated using the Conan Python library. An average occupancy is calculated for three replicates. To measure an interaction we used a distance cutoff of 0.4 nm and a lifetime cutoff of 10%.

References

[1] D. Franke, M. V. Petoukhov, P. V. Konarev, *et al.*, "*ATSAS 2.8* : A comprehensive data analysis suite for small-angle scattering from macromolecular solutions," en, *Journal of Applied Crystallography*, vol. 50, no. 4, pp. 1212–1225, Aug. 2017.

[2] K. M. Ruff and R. V. Pappu, "AlphaFold and Implications for Intrinsically Disordered Proteins," en, *Journal of Molecular Biology*, vol. 433, no. 20, p. 167 208, Oct. 2021.

[3] W. Humphrey, A. Dalke, and K. Schulten, "VMD: Visual molecular dynamics," en, *Journal of Molecular Graphics*, vol. 14, no. 1, pp. 33–38, Feb. 1996.

[4] M. Koi and B. Bertoa, "Trajectory maps: Molecular dynamics visualization and analysis," en, *NAR Genomics and Bioinformatics*, vol. 6, no. 1, lqad114, Jan. 2024.

[5] B. Róycki and E. Boura, "Conformational ensemble of the full-length sars-cov-2 nucleocapsid (n) protein based on molecular simulations and saxs data," *Biophysical Chemistry*, vol. 288, p. 106 843, 2022.

[6] D. Mercadante, F. Gräter, and C. Daday, "Conan: A tool to decode dynamical information from molecular interaction maps," *Biophysical Journal*, vol. 114, no. 6, pp. 1267–1273, 2018.
